# Interpretable metric learning in comparative metagenomics: The adaptive Haar-like distance

**DOI:** 10.1371/journal.pcbi.1011543

**Published:** 2024-05-20

**Authors:** Evan D. Gorman, Manuel E. Lladser

**Affiliations:** Department of Applied Mathematics, University of Colorado, Boulder, Colorado, United States of America; University of Trento, ITALY

## Abstract

Random forests have emerged as a promising tool in comparative metagenomics because they can predict environmental characteristics based on microbial composition in datasets where *β*-diversity metrics fall short of revealing meaningful relationships between samples. Nevertheless, despite this efficacy, they lack biological insight in tandem with their predictions, potentially hindering scientific advancement. To overcome this limitation, we leverage a geometric characterization of random forests to introduce a data-driven phylogenetic *β*-diversity metric, the adaptive Haar-like distance. This new metric assigns a weight to each internal node (i.e., split or bifurcation) of a reference phylogeny, indicating the relative importance of that node in discerning environmental samples based on their microbial composition. Alongside this, a weighted nearest-neighbors classifier, constructed using the adaptive metric, can be used as a proxy for the random forest while maintaining accuracy on par with that of the original forest and another state-of-the-art classifier, CoDaCoRe. As shown in datasets from diverse microbial environments, however, the new metric and classifier significantly enhance the biological interpretability and visualization of high-dimensional metagenomic samples.

## Author summary

Traditional phylogenetic *β*-diversity metrics, particularly weighted and unweighted UniFrac, have had great success in comparing and visualizing high-dimensional metagenomic samples. Nonetheless, these metrics rely upon pre-established biological assumptions that might not capture key microbial players or relationships between some samples. On the contrary, supervised machine learning algorithms, such as random forests, can often capture intricate relationships between microbial samples; however, unveiling these relationships is often challenging due to the intricate inner mechanisms inherent to these algorithms.

The adaptive Haar-like distance integrates the merits of *β*-diversity metrics and random forests, allowing for precise, intuitive, and visual comparison of metagenomic samples, offering valuable scientific insight into the distinctions and associations among microbial environments.

## Introduction

Comparative metagenomics seeks to identify conserved or variable genetic features across microbial communities to discern the relationship between environmental characteristics and microbial composition. A popular approach to infer which microbes are present in a sample is to use amplicon sequencing, targeting the 16S gene, which is present in all bacteria and archaea. The processing of these raw sequences into amplicon reads generates Amplicon Sequence Variants (ASVs), capable of discerning single nucleotide substitutions. ASVs provide a high taxonomic resolution by which to distinguish microbes, offering advantages for study reproducibility among other benefits [1]. Many existing analyses, however, involve a pipeline that clusters these reads (obtained from one or multiple environments) into Operational Taxonomic Units (OTUs) based on a predetermined level of sequence similarity. The OTUs are then consolidated into feature tables with abundance counts per sample. A common practice is to map these OTUs onto the leaves of a reference phylogenetic tree such as Greengenes [2] or Silva [3], allowing for the use of phylogenetic *β*-diversity metrics to quantify differences between microbial environments. If a high resolution of sequence similarity was used to define the OTUs, such as the conventional 97% or 99%, the processed data can have hundreds of thousands of dimensions, which poses significant challenges for its analysis.

In contrast, whole genome sequencing aims to identify all the DNA contained within the microbes’ genome. Traditionally, this process was costly and time-consuming; however, advancements in shotgun sequencing and computational tools have made it feasible. Since this framework does not rely on a singular marker gene, establishing a reference phylogenetic tree is also more challenging. Nevertheless, recent efforts have made strides towards a unified phylogeny for bacterial and archaeal genomes [4].

Phylogenetic *β*-diversity metrics assume that OTUs with shared evolutionary histories possess similar traits, which may be advantageous or disadvantageous in environments with comparable characteristics; in particular, samples containing closely related OTUs should exhibit closer clustering. These metrics are commonly employed to assess the significance of clustering or correlation with covariates such as pH, salinity, or depth [5], among many others. Phylogenetic *β*-diversity metrics are also often used alongside principle coordinates analysis (PCoA) [6] to generate low-dimensional visualizations of microbial datasets [7].

UniFrac [8] is arguably the most renowned phylogenetic *β*-diversity metric. Its fundamental breakthrough lies in effectively integrating microbes’ phylogenetic relatedness: differences in OTU composition between environments are weighted by the shared length of evolutionary history among the OTUs. This metric has two variants, weighted and unweighted. Weighted UniFrac can be viewed as an Earth Mover’s distance, where the ground metric is defined by the underlying reference phylogeny [9].

Double Principle Coordinates Analysis (DPCoA) [10] is another, albeit less well-known, *β*-diversity metric. It is a Mahalanobis-type distance [11] associated with the inverse of the so-called phylogenetic covariance matrix of the reference phylogenetic tree (see Definitions 1.2 and 1.3). This matrix encodes the shared branch length, leading to the root, between all pairs of OTUs [12]. In particular, the entries of this matrix can be interpreted as pair-wise covariances of a trait that evolved over the reference phylogeny according to a Brownian motion [13, Chapter 3].

DPCoA can be considered a Euclidean version (aka *ℓ*^2^-version) of weighted UniFrac, as both metrics rely on the same fundamental assumptions regarding OTU relatedness. (Conversely, UniFrac can be seen as an *ℓ*^1^-version of DPCoA.) While UniFrac and DPCoA and their associated embeddings have demonstrated remarkable efficacy across diverse microbial scenarios, explaining their embeddings solely based on microbial abundances remains a challenge.

Recent work showed that discrete Haar-like wavelets [14] could significantly pseudodiagonalize (i.e., sparsify) the phylogenetic covariance matrix of most large binary trees by changing basis to the wavelets. This motivated the introduction of a novel phylogenetic *β*-diversity metric known as the Haar-like distance [15]. This new metric may be regarded as a proxy for DPCoA; however, unlike DPCoA and UniFrac, it admits a simple decomposition in terms of the splits or bifurcations (i.e., internal nodes) of the reference phylogeny, which enables further interpretation and visualization of microbial sample distances in terms of differences between microbial clade abundances. Despite its breakthrough, the Haar-like distance, along with all existing phylogenetic *β*-diversity metrics, is also constrained by the inherent assumptions in the reference phylogeny (specifically, encoded in its covariance matrix). This bears the question, *can these metrics be tailored to capture more subtle relationships within specific metagenomic datasets, similar to those offered by supervised machine learning techniques, while still leveraging the evolutionary relationships that β-diversity metrics have successfully exploited?*

Random forests (RFs) are a popular supervised machine learning method that combine multiple decision trees to make predictions [16]. While specific architectures may vary among implementations, the fundamental idea is as follows: each tree is built on a random subset of labeled training data and optimizes a criterion such as the Gini impurity [17] to cluster data with similar labels. To make predictions on new unlabeled data, each tree returns a label, and the RF prediction is based on the (potentially weighted) average of these individual tree predictions.

RFs usually exhibit superior or comparative performance to other state-of-the-art methods in microbiome host-trait prediction [18], and numerous studies have documented their effectiveness in metagenomics classification tasks [19–24]. While these classifiers achieve impressive accuracy through the averaging of (random) decision trees, the inherent randomness in their construction can obscure the learned relationships between OTU composition and prediction. Furthermore, RF predictions cannot be explained solely by existing feature importance measures, such as Gini or permutation importance, as they can be highly sensitive to correlated or highly variable features [25, 26].

In this manuscript, we introduce a new phylogenetic *β*-diversity metric: the adaptive Haar-like distance, which is inspired by the recent Haar-like distance [15]. Taking a metric learning approach [27], our algorithm learns a data-dependent weighting of the most important phylogenetic relationships across a set of samples to discover robust representations of microbial abundance patterns. In contrast to traditional metric learning algorithms, which are known to be computationally expensive [27] and suffer from the curse of dimensionality, our approach scales well with large datasets. Scalability is achieved by leveraging a pretrained RF classifier, which we adapt to fit a metric: a Haar-like distance associated with a phylogenetic covariance matrix that we learn from the classifier. Accordingly, the adaptive Haar-like distance combines the predictive power of RFs with the interpretability of the Haar-like distance.

## Materials and methods

In this section, we outline our metric learning algorithm. First, we discuss the Haar-like wavelet [14] basis and its corresponding coordinate system, which gives rise to the Haar-like distance [15]. We then generalize this metric by introducing tunable weight parameters, leading to the adaptive Haar-like distance and corresponding kernel. Then, to learn weights in a data-dependent manner, we examine the representation of random forests as local average estimators [28]. Here, random forest classifiers are framed as kernel estimators built from their so-called affinity. Finally, we use a compressed sensing algorithm to learn a sparse set of weights to approximate a random forest affinity by an adaptive Haar-like kernel. This substitution yields a surrogate random forest model that is precisely interpretable through a limited number of Haar-like coordinates, representing the most relevant clade abundances for distinguishing between environments in a given dataset.

### Haar-like wavelet basis

The Haar-like wavelets were first described in [14] for the multiscale analysis of datasets equipped with a hierarchical partition tree. The wavelets form an orthonormal basis for the vector space of (real-valued) functions defined on the leaves of such trees and localize information on the leaves at scales determined by the proximity of each internal node to the (external) root: the closer an internal node is to the root, the coarser the scale associated with that node.

In phylogenetic trees (particularly out-rooted, see [15, Definition 2.1]), there is a direct correspondence between the Haar-like wavelets and the internal nodes. In particular, since the latter represent speciation events that group OTUs into clades, the Haar-like wavelets offer a basis for comparing clades of microorganisms as opposed to separate OTUs. So, assuming sample abundances correlate within the same clade across similar environments, projecting these onto the wavelets should elucidate relationships between microbial composition and environmental factors [15].

### Haar-like coordinates

Before describing how to project functions defined over the leaves of a phylogenetic tree onto its Haar-like wavelet basis, we introduce some notation.

In what follows, *T* denotes a reference phylogenetic tree with vertex and edge set *V* and *E*, respectively, and branch length function *ℓ*: *E* → [0, + ∞). The root of *T* is denoted as ∘. We distinguish the set of leaves *L* from the set of internal nodes *I*, noting that they partition *V* (i.e., *L* ∪ *I* = *V* but *L* ∩ *I* = ∅). In practice, the leaves of *T* represent OTUs, whereas its interior nodes represent inferred speciation events.

For any internal node *v*, i.e., *v* ∈ *I*, denote by *L*(*v*) the set of leaves that descend from *v*. Further, *v*_+_ and *v*_−_ denote the left and right children descending from *v*, respectively. (In [15], these were denoted *v*0 and *v*1, respectively.)

We assume that microbial abundance data on the leaves of *T* are normalized to sum to one so that each sample can be represented as a probability mass function on *L*. We denote these functions by *x*, *x*_1_, *x*_2_, …; in particular, *x*: *L* → [0, 1) satisfies that ∑_*v* ∈ *L*_*x*(*v*) = 1. Therefore, each sample is compositional (that is, distribution valued) and could be analyzed using a variety of methods [29].

With the above notation, the projection of a sample *x* onto a Haar-like wavelet *φ*_*v*_, associated with internal node *v* ≠ ∘, can be conveniently represented in terms of average abundances on subtrees of the reference phylogeny.

**Definition 1.1** (Average clade size). For a given function x:L→R and non-empty *J* ⊂ *L* of cardinality |*J*|, we define the mean of *x* over *J* as

$$\begin{array}{c} \text{avg}\left(x;J\right)≔\frac{1}{\left|J\right|}\sum_{j\in J}x\left(j\right). \end{array}$$

**Theorem 1.1** (Wavelet projection). Let *v* ≠ ∘ be an interior node of *T*. The projection of a function x:L→R onto Haar-like wavelet *φ*_*v*_ is:

$$\begin{array}{c} \left\langle x,\varphi_{v}\right\rangle =c_{v}\cdot (\text{avg}(x;L(v_{+}))-\text{avg}(x;L(v_{-}))),\;\text{where}\;c_{v}≔\sqrt{\frac{|L\left(v_{+}\right)|\cdot |L\left(v_{-}\right)|}{|L\left(v_{+}\right)|+|L\left(v_{-}\right)|}}. \end{array}$$

We refer to the set of projections {〈*x*, *φ*_*v*_〉}_*v* ∈ *I*\{∘}_ as the **Haar-like coordinates** associated with a sample *x*. (We disregard the root of *T* in our setting because, for compositional data *x*, $\langle x,\varphi_{{}^{\circ}}\rangle =\sqrt{\left|L\right|}$, i.e., a constant. In particular, as we assess microbial samples by differences in their Haar-like coordinates, this coordinate holds no relevance in our framework.)

We highlight that the Haar-like coordinates of log(*x*) (i.e., the function log(*x*(*v*)), when *x*(*v*) > 0 for all *v* ∈ *L*) correspond to the isometric log-ratio (ILR) coordinates [30], which have been used in previous metagenomics analyses like PhILR [31] and Phylofactorization [32]. The ILR coordinates necessitate zero-count replacement and employ logarithmic ratios of geometric means to map compositional data into an unconstrained Hilbert space, known as the Bayes space [33], where the Euclidean distance is replaced by the Aitchison distance [34]. The Aitchison distance is invariant to the underlying phylogenetic structure and thus disregards OTUs evolutionary relatedness, which has been key to the success of phylogenetic *β*-diversity metrics.

### Haar-like distance

As mentioned earlier, DPCoA is a Mahalanobis-type distance associated with the inverse of the phylogenetic covariance matrix of the reference tree. The precise interpretations of this statement follow.

**Definition 1.2** (Phylogenetic Covariance). For *i*, *j* ∈ *V*, let [*i*, *j*] denote the set of edges in the shortest path between nodes *i* and *j* in *T*. Also, let (*i* ∧ *j*) be the least common ancestor of *i* and *j*. Namely, the *v* ∈ *V* that maximizes |[*v*, ∘]| among all the nodes that are ancestors to both *i* and *j*. The phylogenetic covariance matrix of *T* is the matrix of dimensions |*L*| × |*L*| with entries

$$\begin{array}{c} C\left(i,j\right)≔\sum_{e\in [i\wedge j,\circ ]}\ell \left(e\right),\;\text{for}\;\text{each}\;i,j\in L. \end{array}$$

In what follows, *A*^*T*^ denotes the transpose of a vector or matrix *A*.

**Definition 1.3** (Double Principal Coordinate Analysis [10]). The DPCoA distance between two environmental samples *x*_1_ and *x*_2_ is

$$\begin{array}{c} \text{DPCoA}\left(x_{1},x_{2}\right)≔\sqrt{{\left(x_{1}-x_{2}\right)}^{T}C\left(x_{1}-x_{2}\right)}. \end{array}$$ (1)

Let Φ denote the matrix whose columns consist of the Haar-like wavelets of the reference phylogeny. On large trees, if one changes basis using Φ, then, in the new coordinates, and with high probability, *C* will be nearly diagonal [15, Corollary 3.8]. Namely, the matrix Φ^*T*^ *C*Φ is significantly sparse, which motivates substituting *C* by the diagonal matrix

$$\begin{array}{c} \Lambda ≔\text{diag}(\lambda_{v}:v\in I), \end{array}$$

where

$$\begin{array}{c} \lambda_{v}≔\left(\Phi^{T}\;C\;\Phi \right)\left(v,v\right)=\varphi_{v}^{T}C\varphi_{v},\;\text{for}\;\text{each}\;v\in I. \end{array}$$

In terms of DPCoA, this is equivalent to substituting the matrix *C* in (1) by the matrix ΦΛΦ^*T*^, which motivates the next definition.

**Definition 1.4** (Haar-like Distance [15]). The Haar-like distance between two environmental samples *x*_1_ and *x*_2_ is

$$\begin{array}{c} d\left(x_{1},x_{2}\right)≔\sqrt{{\left(x_{1}-x_{2}\right)}^{T}\Phi \Lambda \Phi^{T}\left(x_{1}-x_{2}\right)}=\sqrt{\sum_{v\in I}\lambda_{v}\;{\left\langle x_{1}-x_{2},\varphi_{v}\right\rangle }^{2}}. \end{array}$$ (2)

The Haar-like distance is a weighted Euclidean distance between the Haar-like coordinates of pairs of samples. This metric provides an interpretable version of DPCoA, as the distance between two samples relates to a sum indexed by the internal nodes of the reference phylogeny and, unlike the Aitchison distance, includes assumptions about phylogenetic relatedness when calculating distances.

### Adaptive Haar-like distance and kernel

Although traditional phylogenetic *β*-diversity metrics have provided significant insights across various datasets, they may not consistently differentiate between samples from distinct environments. On the other hand, despite its biological interpretability, a fundamental limitation of the newly introduced Haar-like distance is the potentially broad biological assumptions encoded by the coefficients λ_*v*_, with *v* ∈ *I*, used to define it (see Eq (2)). In fact, it seems improbable that these fixed “universal” weights adequately account for relevant differences in microbial composition across arbitrary pairs of environments. Nevertheless, the Haar-like distance allows for easy adjustment of these assumptions by replacing its fixed weights with adaptive ones, learned from labeled datasets. We next define this generalization.

**Definition 1.5** (Adaptive Haar-like Kernel & Distance). The adaptive Haar-like kernel associated with a weight vector *w* = {*w*_*v*_}_*v* ∈ *I*_, with *w*_*v*_ ≥ 0 for each *v*, is defined as

$$\begin{array}{c} k_{w}\left(x_{1},x_{2}\right)≔\langle Wx_{1},Wx_{2}\rangle ,\;\text{where}\;W≔\text{diag}(\sqrt{w})\Phi^{T}. \end{array}$$ (3)

The adaptive Haar-like distance between two environmental samples *x*_1_ and *x*_2_ is

$$\begin{array}{c} d_{w}\left(x_{1},x_{2}\right)≔\sqrt{k_{w}\left(x_{1}-x_{2},x_{1}-x_{2}\right)}. \end{array}$$ (4)

The adaptive Haar-like distance is induced by an inner product between differences of Haar-like coordinates; in particular, it is faithful to the topology of the reference tree. Importantly, each weight aligns with an internal node in the phylogeny, allowing selective weight adjustment for specific clades. This is especially promising for a multiscale analysis of *β*-diversity in sample comparisons. In practice, however, identifying the most important internal nodes in a given context is not immediately clear. The subsequent aim is therefore to choose weights that minimize the adaptive Haar-like distance, or maximize the related kernel, between samples that share similar environmental characteristics.

In the framework of kernel regression [35], the Haar-like kernel could be used to construct an estimator $\hat{y}$ of the data labels $y:R^{d}\mapsto C\subset R$. Subsequently, finding the optimal weights for a given dataset of *n* labeled samples (*x*_1_, *y*_1_), …, (*x*_*n*_, *y*_*n*_) could be achieved through optimization of the quadratic leave-one-out training loss with respect to the vector of weights *w*:

$$\begin{array}{c} \underset{w}{\text{arg}\;\text{min}}\sum_{i=1}^{n}{\left(y_{i}-{\hat{y}}_{i}\right)}^{2},\;\text{where}\;{\hat{y}}_{i}={\hat{y}}_{i}\left(x;w\right)≔\frac{\sum_{i\neq j}^{n}\;k_{w}\left(x,x_{i}\right)\cdot y_{i}}{\sum_{i\neq j}^{n}\;k_{w}\left(x,x_{i}\right)}. \end{array}$$ (5)

In theory, this optimization could be done using gradient descent as in [36]. Unfortunately, the cost to compute the gradient **in each iteration** is $O(dn^{2})$, which is prohibitively expensive in high dimensions *d*. Instead, we introduce a method to efficiently infer weights from a pre-trained random forest (RF) that requires only a **single** $O(dn^{2})$ computation.

### Towards an interpretable random forest surrogate

Though the connection to metric learning is not immediately clear, an RF can be re-framed as a kernel method by considering the geometry learned through training: each decision tree is a collection of binary decision rules that partition a feature space. By examining the splits made by the decision trees, it is possible to define a notion of similarity between data points based on the trees’ paths they traverse within the forest.

The RF affinity [28] is a kernel that quantifies how often two data points land in the same partition across a forest’s decision trees. This kernel can be used to replicate RF predictions: the label for a new point is estimated through a weighted average of the closest training point labels based on some similarity measure.

In what follows, ⟦⋅⟧ denotes the indicator function (aka Iverson bracket) of the proposition within, i.e., ⟦⋅⟧ = 1 when the statement within parenthesis is true; otherwise ⟦⋅⟧ = 0.

**Definition 1.6** (Random Forest Affinity [28] & Dissimilarity). Consider an RF consisting of *M* decision trees trained on *n* labeled samples $\left(x_{i},y_{i}\right)\in \left(R^{d},C\right)$, where C⊂R is non-empty. (For example, C={-1,1} in binary classification, but C=R in most regression problems.) For each $x\in R^{d}$, let $L_{m}\left(x\right)$ denote the bin containing *x* in the *m*-th decision tree. The RF affinity and dissimilarity between two points $x_{1},x_{2}\in R^{d}$ are defined, respectively, as follows:

$$\begin{array}{cc} A(x_{1},x_{2}) & ≔\frac{1}{M}\sum_{m=1}^{M}\left[\;\left[L_{m}\left(x_{1}\right)=L_{m}\left(x_{2}\right)\right]\;\right]; \end{array}$$ (6)

$$\begin{array}{cc} D(x_{1},x_{2}) & ≔1-A(x_{1},x_{2}). \end{array}$$ (7)

The RF affinity between two points is the fraction of decision trees that group them in the same leaf across the forest; in particular, it is symmetric and measures how similar two points are from the perspective of the trained RF. Accordingly, the dissimilarity is also symmetric but measures how often two training points are placed into different bins by the RF.

In the context of regression, the affinity can be used to construct the so-called kernel RF estimate from labeled samples (*x*_1_, *y*_1_), …, (*x*_*n*_, *y*_*n*_), as follows.

**Definition 1.7** (RFs as Regressive Local Average Estimators [28]). The kernel RF estimate (KeRFE) of a function $f:R^{d}\to R$ at a point *x* is

$$\begin{array}{c} \hat{f}\left(x\right)≔\frac{\sum_{i=1}^{n}\;A\left(x,x_{i}\right)\cdot y_{i}}{\sum_{i=1}^{n}\;A\left(x,x_{i}\right)}. \end{array}$$

Under mild conditions, the KeRFE converges to the original RF estimate as *n* increases [28].

KeRFEs do not entirely resolve the interpretability issue of RFs. One reason is the non-stationarity of *A*(*x*_1_, *x*_2_) [37], as it depends on both *x*_1_ and *x*_2_ rather than just (*x*_1_ − *x*_2_), which complicates generating a consistent explanation of the affinity’s behavior across a dataset. This is unlike the adaptive Haar-like kernel and distance, where weights signal the importance of clade abundances in sample comparisons. Nevertheless, as we shall see next, KeRFEs offer a perspective for directly studying the behavior of RFs through their affinity.

### Metric learning algorithm

**The core idea in this manuscript** is to learn a weight vector *w* such that the Haar-like kernel (see Eq (3)) can act as a surrogate of the RF affinity (see Eq (6)) across the whole training set. In particular, because each weight *w*_*v*_ is directly linked to the internal node *v* and, therefore, a speciation event in the reference tree, the associated Haar-like kernel may serve as an interpretable proxy for the RF model—from the perspective of the phylogeny. We note that after learning the appropriate weights, the adaptive Haar-like distance and its associated embedding can be recovered from the kernel.

Assume as given *n* labeled samples $\left(x_{i},y_{i}\right)\in \left(R^{d},C\right)$ and collect the *x*_*i*_’s in a data matrix $X\in R^{d\times n}$. To accomplish our goal, we first train an RF on the data and recover a pairwise affinity matrix $A\in R^{n\times n}$ and dissimilarity matrix *D* ≔ **1** **1**^*T*^ − *A*, where **1** is a column vector of ones of dimension *n*. (The entry in row-*x*_*i*_ and column-*x*_*j*_ of *A* and *D* are *A*(*x*_*i*_, *x*_*j*_) and *D*(*x*_*i*_, *x*_*j*_), respectively.)

Although *D* is in general non-Euclidean, we can use principal coordinate analysis (PCoA), also known as multidimensional scaling [38], to find a matrix *Z* such that the Euclidean distance between its *i*-th and *j*-th column is approximately equal to *D*(*i*, *j*). Therefore, the matrix *G* ≔ *Z*^*T*^*Z* is of a Gram-type [39] as its entries are the Euclidean inner products between all the columns in *Z*. In practice, we find that this Euclidean approximation of *D* has no noticeable effect on the resulting model performance (see Fig E in S1 Text).

**Fig 1:**
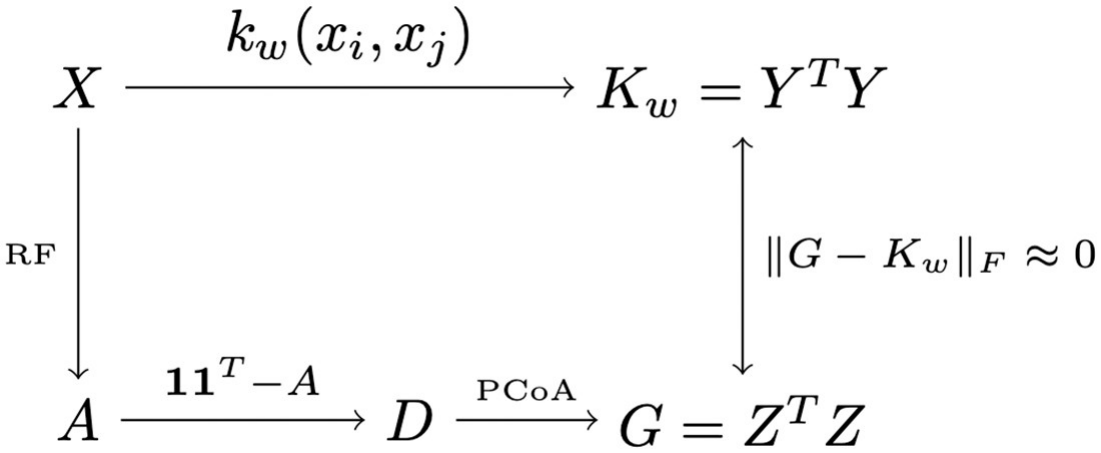
Illustration of the main mathematical objects and their relationships in our metric learning algorithm.

Define *K*_*w*_ ≔ *X*^*T*^Φ diag(*w*)Φ^*T*^*X*; in particular, the entry associated with row-*x*_*i*_ and column-*x*_*j*_ of this matrix is precisely *k*_*w*_(*x*_*i*_, *x*_*j*_). The matrix *K*_*w*_ like *G* is also of a Gram-type because *K*_*w*_ = *Y*^*T*^*Y*, with $Y≔\text{diag}(\sqrt{w})\Phi^{T}X$. Ideally, we would like to select a weight vector *w* so that *K*_*w*_ = *G*; however, this is not generally possible. So instead, we pursue the next best option: find a vector *w* such that *K*_*w*_ approximates *G* as best as possible, which we interpret as solving the optimization problem:

$$\begin{array}{c} \underset{w\in R^{d}}{\text{min}}∥G-K_{w}{∥}_{F},\;\text{subject}\;\text{to}\;{∥w∥}_{0}\leq s, \end{array}$$ (8)

where ‖⋅‖_0_ is the pseudo-norm that counts the number of nonzero entries of the vector. The inclusion of the constraint ‖*w*‖_0_ ≤ *s*, where *s* is a strictly positive user-defined integer, ensures that the optimization prioritizes sparse solutions, thereby enhancing the interpretability of the solution *w*. Fig 1 gives an overview of the approach we have just described.

It is worth noting that the identities, *G* = *Z*^*T*^*Z* and *K*_*w*_ = *Y*^*T*^*Y*, prompt the approximation of Euclidean coordinates in *Z* by those in *Y*. Nevertheless, this alternative approach is unsuitable because it is not rotation-invariant, unlike the formulation based on Gram matrices in (8).

For any matrix *M*, let *M*_*i*_ denote its *i*-th column and vec(*M*) be the (column) vector obtained by stacking *M*_1_, *M*_2_, … up from left to right.

We can reformulate the optimization problem in (8) into a more computationally tractable one as follows. Since *K*_*w*_ is linear in *w*, there is a matrix $M\in R^{n^{2}\times d}$ such that vec(*K*_*w*_) = *Mw*. In particular, since ‖*G* − *K*_*w*_‖_*F*_ = ‖vec(*G*) − vec(*K*_*w*_)‖_2_, the minimization problem is equivalent to

$$\begin{array}{c} \underset{w\in R^{d}}{\text{min}}{∥\text{vec}\left(G\right)-Mw∥}_{2},\;\text{subject}\;\text{to}\;{∥w∥}_{0}\leq s. \end{array}$$ (9)

Further, the columns of *M* can be computed explicitly as follows. Let *e*_*i*_ denote the *i*-th vector in the canonical base of $R^{d}$; namely, *e*_*i*_(*j*) = ⟦*j* = *i*⟧ for 1 ≤ *j* ≤ *d*. Then, we must have $M_{i}=\text{vec}\left(K_{e_{i}}\right)=\text{vec}\left(X^{T}\Phi \text{diag}\left(e_{i}\right)\Phi^{T}X\right)$. But diag(*e*_*i*_) is symmetric and idempotent; hence

$$\begin{array}{c} M_{i}=\text{vec}({\left(\text{diag}\left(e_{i}\right)\Phi^{T}X\right)}^{T}\text{diag}\left(e_{i}\right)\Phi^{T}X)=\text{vec}({\left(X^{T}\Phi \right)}_{i}⊗{\left(X^{T}\Phi \right)}_{i}), \end{array}$$

where ⊗ denotes the outer-product of vectors. Namely, *M*_*i*_ is the vectorization of the matrix obtained by the outer-product of the *i*-th row of Φ^*T*^*X* with itself. We emphasize that Φ^*T*^*X* is the matrix of Haar-like coordinates of the (unlabelled) data.

The optimization in Eq (9) is a standard sparse approximation problem [40], where the matrix *M* is referred to as the “dictionary,” and the goal is to learn a sparse linear combination of the “dictionary elements” (i.e., columns of *M*) to best reconstruct a signal. In our setting, **in a dataset-specific manner**, the signal is the matrix *G*, which is a proxy for the discriminatory patterns learned by the RF, whereas *M*’s columns represent discriminatory patterns associated with the internal nodes of the reference phylogeny. **The goal of the sparse approximation in (9) is therefore to find the least number of Haar-like coordinates that can best explain the patterns learned by the RF**.

In general, the minimization problem in (9) is NP-hard, but there exists a variety of approaches to find an approximate solution. One popular formulation, basis pursuit denoising [40], relaxes the ||⋅||_0_ pseudo-norm constraint using an ||⋅||_1_-norm regularizer. This is then a convex quadratic problem for which many solvers exist [41, 42]. However, with interpretability in mind, we would like full control over the sparsity of our solution. Hence we opt to instead approximate the solution to (9) using Algorithm 1, a heuristic variant of the Matching Pursuit (MP) algorithm [40], in which the weights are constrained to be non-negative.

**Algorithm 1 Non-negative Matching Pursuit Algorithm**

**Input**. *G*, *M*, *s*.

**Output**. *ι*: {1, …, *s*}→{1, …, *d*}, with *d* = |*I*|, and v:{1,…,s}→R.

*R*_1_ ← vec(*G*)

**while** there exists at least one positive inner product $\langle R_{i},\frac{M_{j}}{∥M_{j}∥}\rangle$ and *i* ≤ *s* **do**

 $\iota \left(i\right)\leftarrow \underset{j=1:d}{\text{arg}\;\text{max}}\langle R_{i},\frac{M_{j}}{∥M_{j}∥}\rangle$

 *v*(*i*) ← 〈*R*_*i*_, *M*_*ι*(*i*)_〉

 *R*_*i*+1_ ← *R*_*i*_ − *v*(*i*)*M*_*ι*(*i*)_

 *i* = *i* + 1

Algorithm 1 takes as input the signal *G*, the dictionary *M*, and a user-defined sparsification parameter *s*. It returns functions *ι*: {1, …, *s*}→{1, …, *d*} and v:{1,…,s}→R such that the vector *w* with zero entries, except that *w*(*ι*(*i*)) = *v*_*i*_ for *i* = 1: *s*, approximately solves the optimization problem in (9). The algorithm is greedy, which lets us choose exactly how sparse of a solution we want. Its key idea is to select iteratively the dictionary element with the largest projection along the signal, subtract this projection from the signal, and then repeat with the signal residual.

In principle, all inner products may be negative in the first iteration of the algorithm, in which case it will return no weights. Otherwise, as in traditional MP, the same column may be selected more than once. Nevertheless, we did not observe any of these anomalous behaviors when the sparsity was constrained to 10 or fewer coordinates.

The function *v* need not be decreasing, i.e., *v*(*i*) may be larger than *v*(*i*+ 1). Nevertheless, the norm of *R*_*i*_ − *R*_*i*+1_ = *v*(*i*)*M*_*ι*(*i*)_ is a decreasing function of *i* (this follows from the non-constrained version of Matching Pursuit [43]) and a natural measure of the importance of the Haar-like coordinate with index *ι*(*i*); accordingly, we refer to |*v*(*i*)|⋅‖*M*_*ι*(*i*)_‖ as a **Haar-like coordinate importance**.

Finally, because the learned weights *w*_*v*_, with *v* ∈ *I*, are constrained to be non-negative, the resulting Gram matrix, *K*_*w*_ = *X*^*T*^*W*^*T*^*WX*, with $W≔\text{diag}(\sqrt{w})\Phi^{T}$ (see Definition 3), can be factored to find an associated Euclidean embedding with coordinates given by *WX*.

### The Haar-like kernel as a local average estimator

The question remains: **how consistent is the adaptive Haar-like surrogate model with the original random forest?**

In this section, we detail how the adaptive Haar-like kernel can be used as a local average estimator, similar to the KeRFE, to obtain estimates of unlabelled data points. Later, this allows us to benchmark our metric against the original random forest and another interpretable model, CoDaCoRe [44].

Again consider *n* labeled samples $\left(x_{i},y_{i}\right)\in \left(R^{d},C\right)$ with {*x*_*i*_}_*i* = 1: *n*_ collected into a data matrix $X\in R^{d\times n}$. We train the random forest using these labeled samples. Suppose we are then given *m* new unlabelled samples ${\left\{x_{i}\right\}}_{i=n+1}^{n+m}$ that are appended to the data matrix to form $\tilde{X}\in R^{d\times (n+m)}$. First, we construct estimates for these new points using the trained random forest. We then recover the random forest affinities between **all** points to construct the full affinity matrix ${\tilde{A}}_{RF}\in R^{\left(n+m)\times (n+m\right)}$. (Recall that affinity matrices are symmetric.) Next, we apply the metric learning algorithm to this affinity matrix to recover the Gram matrix *K*_*w*_ and associated Haar-like coordinates $W\tilde{X}$. The Euclidean distances between these points are computed to form a Euclidean distance matrix *D*_Haar_. Values in this matrix are threshold to a maximum of one, allowing us to form the Haar affinity: *A*_Haar_ = 1 − *D*_Haar_. Using this learned Haar affinity, the surrogate estimate for the RF is:

$$\begin{array}{c} {\hat{f}}_{\text{Haar}}\left(x_{n+1}\right)=\frac{\sum_{i=1}^{n}\;A_{\text{Haar}}\left(x_{n+1},x_{i}\right)y_{i}}{\sum_{i=1}^{n}\;A_{\text{Haar}}\left(x_{n+1},x_{i}\right)}. \end{array}$$ (10)

By replacing the RF affinity with our Haar-like affinity, we now have constructed an **interpretable** surrogate for the RF estimator: estimates are made by comparing to neighbors, and neighbors are determined by comparing the learned Haar-like coordinates. Ahead, we demonstrate that this surrogate has comparable performance to the original RF.

## Results

In this section, our goals are twofold: to demonstrate the use of the adaptive Haar-like distance as an exploratory tool in metagenomics datasets (**Model Demonstration**), and to verify that our model is a suitable approximation of the original random forest (**Model Validation**).

For the model demonstration, we apply the adaptive Haar-like distance to five datasets spanning categorical and continuous labels across varied biological settings (see Table 1). We examine the learned Haar-like coordinates for each dataset, producing visualizations and associating them with known biological contexts. Next, for the validation, we benchmark the adaptive Haar-like distance classification performance against the standard RF and another interpretable classifier, CoDaCoRe [44].

**Table 1:** Datasets used for model demonstrations.

Dataset	Task	Sample Type	Sample Count	No. of Classes	Sequencing Method
Costello et. al. 2009 [45]	Body Site (classification)	Various Body Sites	600	7	16S
Dan et. al. 2020 [46]	Autism Spectrum Disorder (classification)	Human Fecal	286	2	16S
Youngblut et. al. 2020 [47]	Animal Diet (classification)	Animal Gut	628	4	WGS
Mills et. al 2019 [48]	Calprotectin Levels (regression)	Human Fecal	24	n/a	WGS
Mason et. al. 2014 [49]	Distance from Wellhead (regression)	Ocean Sediment	106	n/a	16S

Our analyses include both 16S rRNA sequence data and whole genome sequence (WGS) data. For the 16S datasets, we use Greengenes 97% [2] as the reference phylogenetic tree with associated taxonomy from NCBI [51]. For the WGS datasets, we use the Web of Life (WoL) phylogeny [4] annotated by the GTDB [52] taxonomy. All datasets and phylogenetic trees used in this manuscript were obtained through QIITA [53].

Due to the correspondence between the Haar-like basis and the set of internal nodes in the reference phylogenetic tree, we can construct a helpful visualization for the learned metric over a dataset. In particular, we can assemble **phylogenetic spectrograms**, which shade clades in the reference phylogeny in order of importance of their learned weights (see Definition 1.5). We generate spectrograms using iTOL [54].

Our general methodology has two user-controlled tuning parameters: the sparsity *s* (i.e., the number of Haar-like coordinates to recover), and the minimum RF bin size (i.e., the minimum number of samples for a node to be considered a leaf in the original RF), initially set during the training of the original RF. In the classification setting, we always set the minimum RF bin size to 1 for optimal performance. However, in the regression setting, too small a bin size may reduce the RF affinity between similar samples and can make it more difficult for our algorithm to recover clustering patterns. Accordingly, we set the bin size at the ceiling of 10% of the total sample count, noting that optimal bin size may require further experimentation.

### Model demonstration

The purpose of this section is to introduce the adaptive Haar-like distance as an exploratory tool to **link differences in environmental characteristics (given by sample labels) to variations in clade abundances**. We show that across a diverse range of microbial environments, our metric produces embeddings that display strong clustering (in the classification setting) and strong gradients (in the continuous setting) with respect to these sample labels.

A particularly useful aspect of our metric is that, due to Defition 1.5, the Haar-like coordinates can be treated as Euclidean ones. These coordinates correspond to speciation events in the reference phylogenetic tree, facilitating direct visualization of the relationship between changes in clade abundances and directions within the embedding through a biplot [55], where loadings are associated with Haar-like coordinates. Notably, by constraining the number of Haar-like coordinates, we achieve a distinct advantage over existing phylogenetic *β*-diversity metrics: the resulting embedding can be explained **exactly** by a small number of clades.

We underscore that our aim here is not to replicate a full scientific analysis of these datasets but to demonstrate that our metric recovers Haar-like coordinates associated with biologically significant clades. With this in mind, for each dataset, we select ahead of time a sparsity parameter *s* and link the top *s* Haar-like coordinates to established taxonomical annotations by identifying the lowest taxonomic classification that encompasses all members of the corresponding clade. While further Haar-like coordinates may be relevant—depending on the dataset—we limit our discussion to these top *s* coordinates. Nevertheless, an in-depth analysis of the relevant Haar-like coordinates should pay close attention to the comparison of abundances of the left and right descendants of the corresponding internal nodes. For instance, an observed increase in a Haar-like coordinate does not necessarily imply increased abundances of all its descendants. Instead, recall from Theorem 1.1 that the Haar-like coordinate values represent the difference between the abundances of left and right subtrees.

### Dataset 1 classification: Body sites

We use the first dataset as a detailed exposition to our method, thoroughly explaining all related plots. Our analysis becomes more succinct for subsequent datasets, focusing solely on the most critical observations.

This dataset consists of 16S rRNA sequences from “Bacterial community variation in human body habitats across space and time” [45]. This study “surveyed bacteria from up to 27 sites in seven to nine healthy adults on four occasions” resulting in a total of 600 samples. For our analysis, we grouped these into 7 primary body habitats: skin, external auditory canal, feces, hair, oral cavity, nostril, and urine.

**Fig 2:**
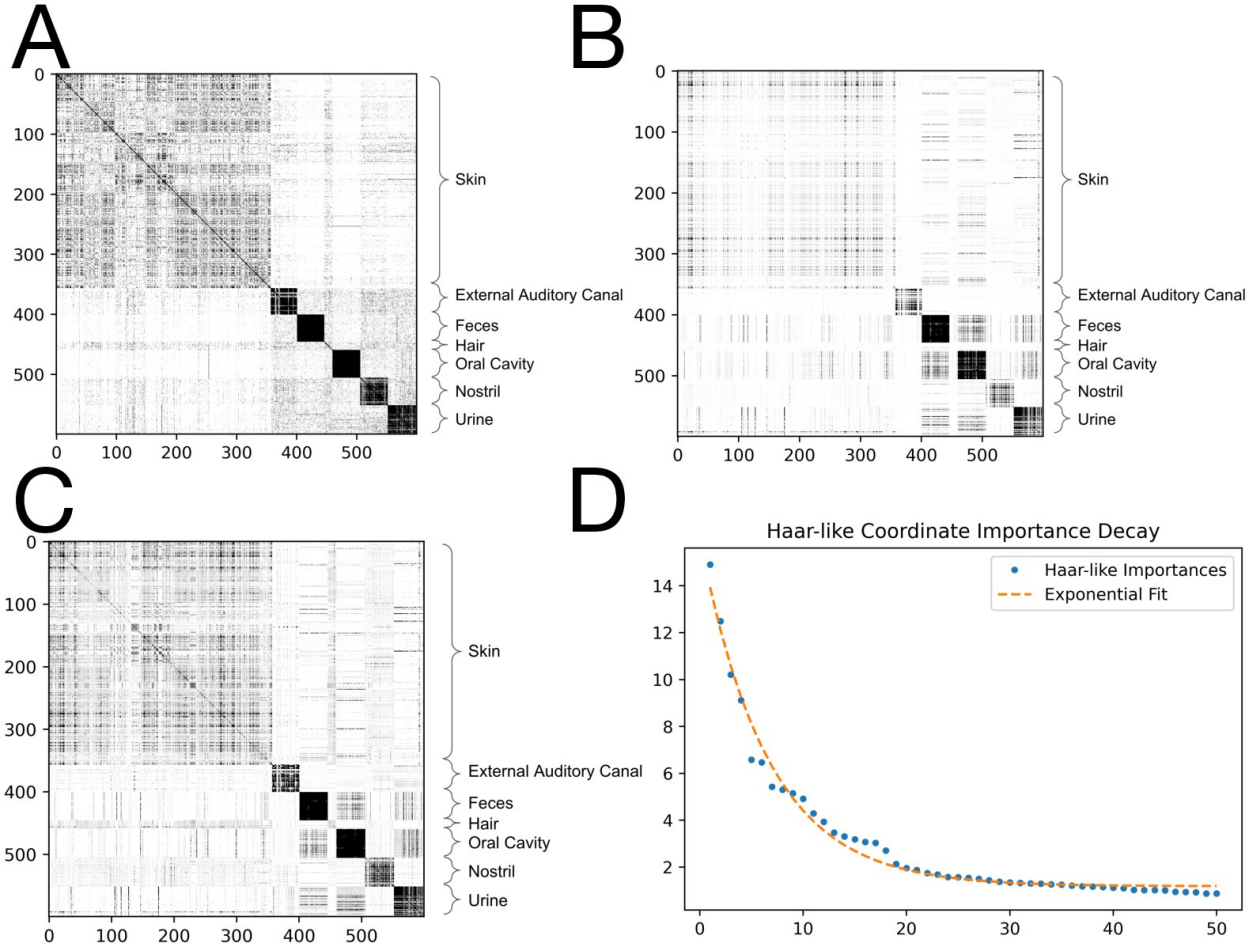
Sparse approximation of the RF Gram matrix from the Body Sites dataset. A: RF Gram matrix. B: Sparse approximation using 7 Haar-like coordinates. C: Sparse approximation using 50 Haar-like coordinates. D: Haar-like coordinate importance as learned by Algorithm 1. The fit is *y* = 13.69*e*^−.14*x*^ + 1.09.

Training the RF on all 600 labeled data points, we form the RF Gram matrix *G* shown in Fig 2A. Here, the indices have been sorted by body habitat. For this dataset, motivated by the fact that there are seven body habitats, we first applied the non-negative Matching Pursuit algorithm with *s* = 7 to recover the seven most important Haar-like coordinates. The associated Gram matrix *K*_*w*_ constructed from these coordinates is shown in Fig 2B. We also display the Gram matrix resulting from the first 50 coordinates in Fig 2C. In both cases, we find a good reconstruction of the true RF affinity with only a small amount of additional noise. Fig 2D displays the importance of these top 50 Haar-like coordinates. We note that the exponential decay of these importances implies low dimensional embeddability of the data and indicates the efficiency of our adaptation of Matching Pursuit (Algorithm 1) in choosing relevant Haar-like coordinates.

**Fig 3:**
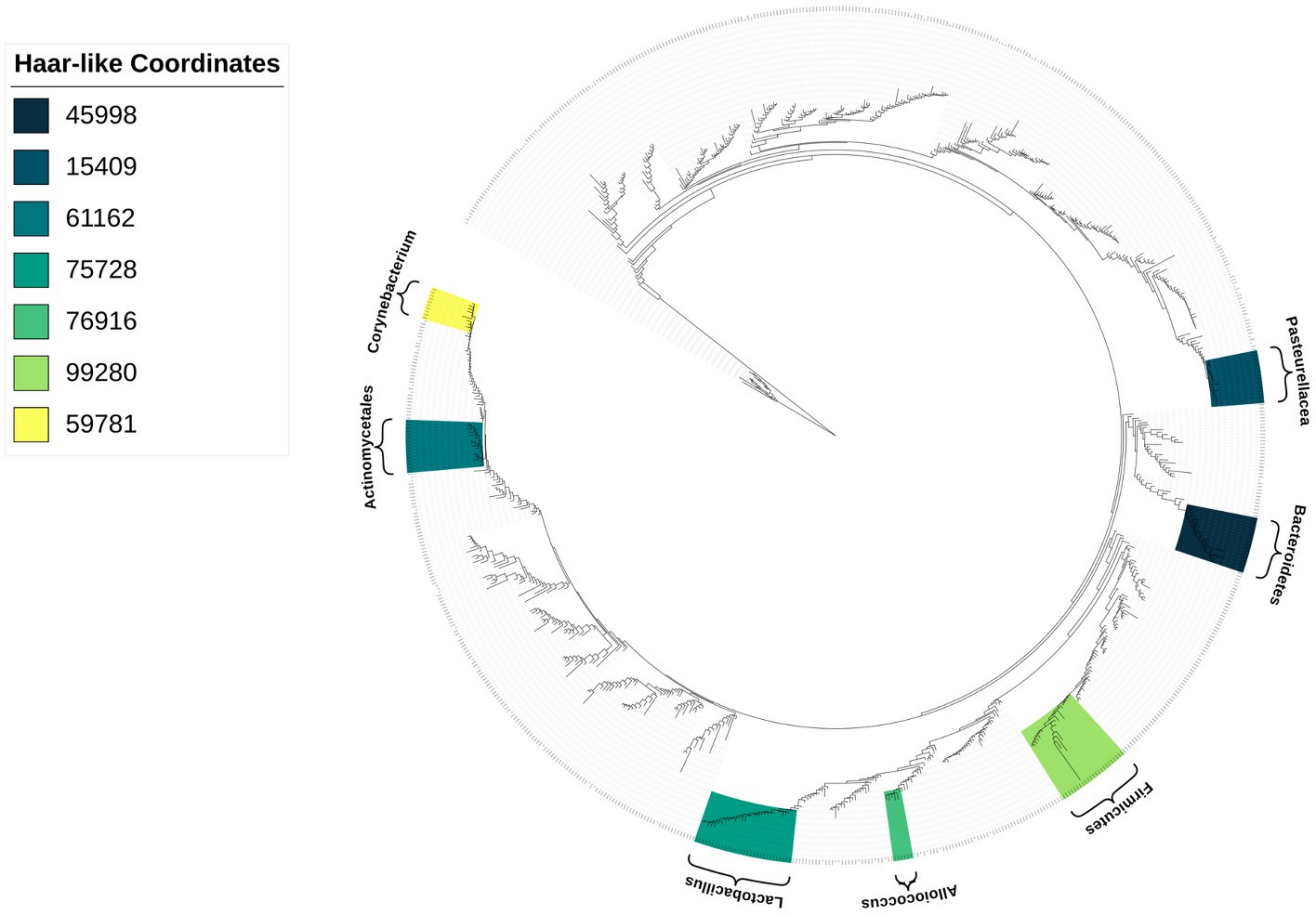
The seven most important Haar-like coordinates of the Body Sites dataset visualized on Greengenes 97%. Colors are displayed in decreasing order of importance from darker to lighter shades.

**Fig 4:**
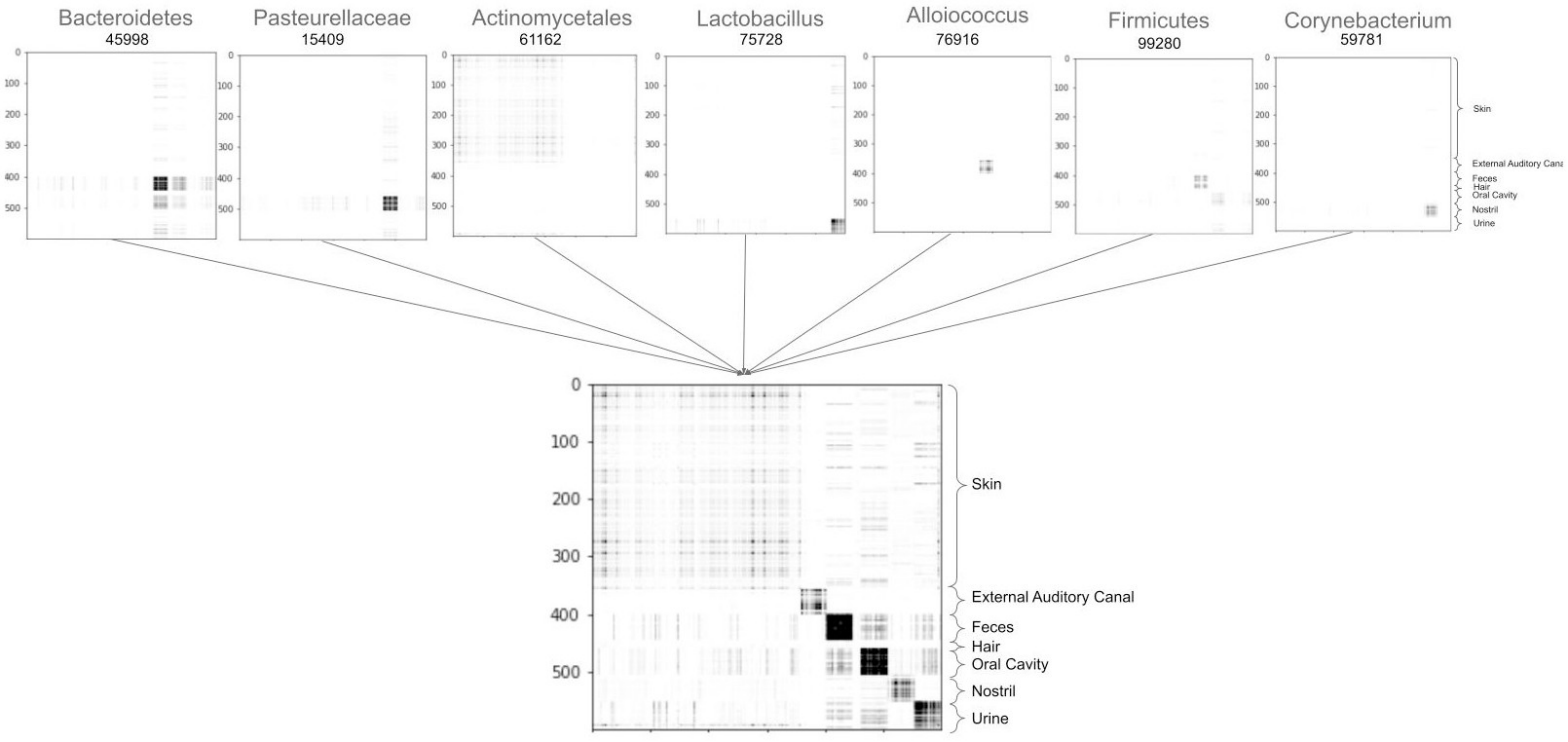
Reconstructed RF Gram matrix of the Body Sites dataset using the seven most dominant Haar-coordinates. These have indexes 45998, 15409, 61162, 75728, 76916, 99280, and 59781 in a post-order traversal of Greengenes 97%.

Fig 3 displays the phylogenetic spectrogram associated with the top seven Haar-like coordinates of the Body Sites dataset. Moreover, for illustration, Fig 4 displays how these Haar-like coordinates combine to capture the vast majority of the classification pattern (excluding hair samples) seen in the RF Gram matrix (Fig 2B). We note that hair samples make up only about ∼2% of the dataset, so their lack of distinction with only seven Haar-like coordinates is not surprising.

**Fig 5:**
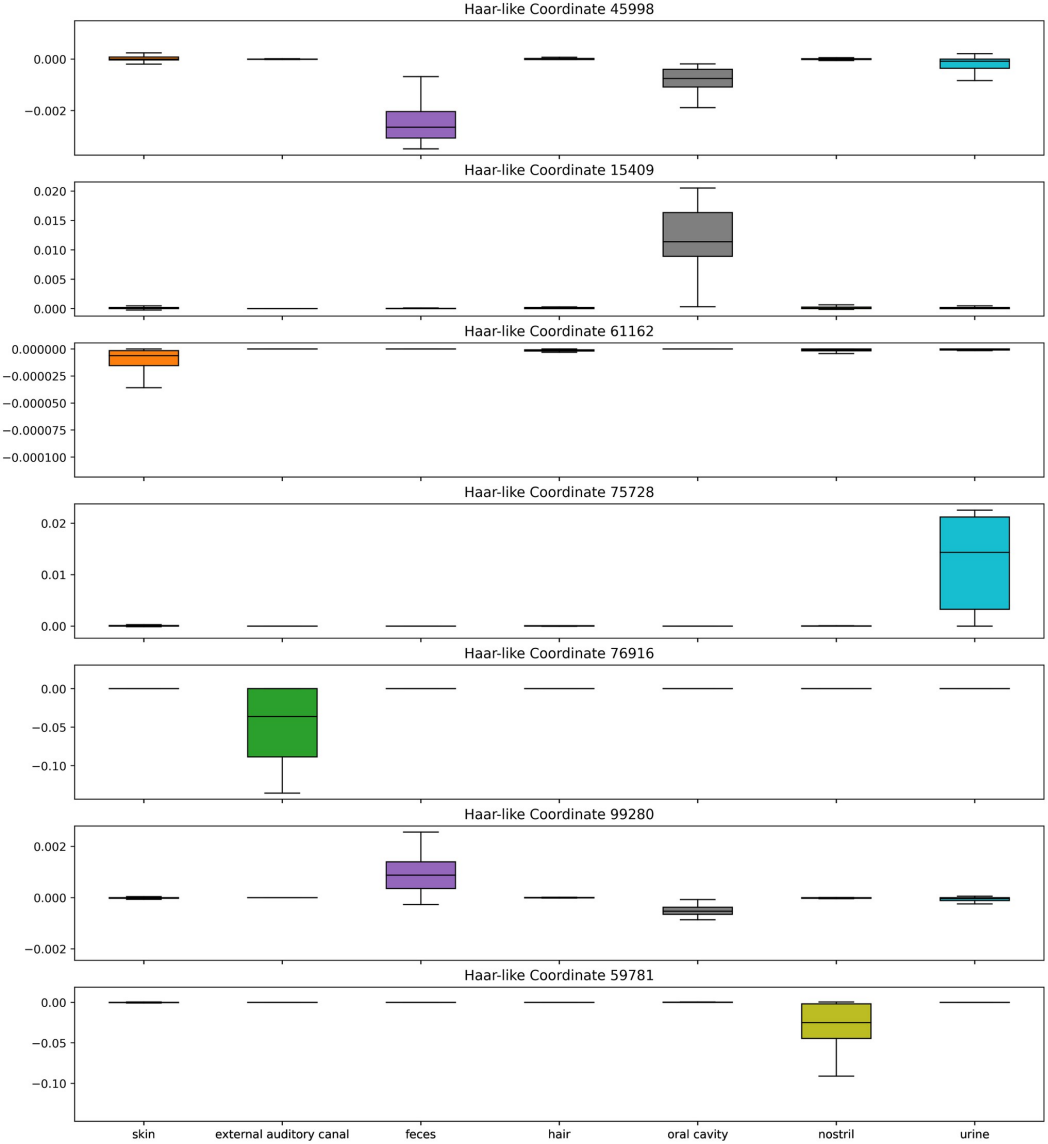
Box plots of the top seven Haar-like coordinates across the Body Sites dataset.

To confirm that our algorithm is recovering biologically meaningful splits in Greengenes 97%, we further examine these first seven selected Haar-like coordinates to assess their relevance to the habitat of interest. To aid in our analysis, Fig 5 displays boxplots of these seven coordinates in the different body habitats.

The dominant Haar-like coordinate (45998) strongly localizes fecal samples (with a negative coordinate value) and also corresponds, to a lesser extent, with oral cavity and urine samples. Notably, the descendants of node 45998 are classified as Bacteroidetes, a phylum well known to be found in the human gut, but also in the mouth and urine [56].

The second selected Haar-like coordinate (15409) localizes oral cavity samples. On examination of the phylogeny, this clade consists entirely of the Pasteurellaceae family, which has been identified in human supragingival plaque samples [57].

The third selected Haar-like coordinate (61162) corresponds to the order Actinomycetales. As seen in Fig 5, this coordinate strongly localizes the skin samples. The literature supports that Actinomycetales, specifically the order genus Actinomyces, appear in the skin [58].

The fourth selected Haar-like coordinate (75728) localizes urine samples and consists entirely of the genus Lactobacillus, which has been found in both male and female urine [59, 60].

The fifth selected Haar-like coordinate (76916) localizes the external auditory canal and consists entirely of the genus Alloiococcus, which has been established as part of the typical outer ear microbiome [61].

The sixth selected Haar-like coordinate (99280) again localizes fecal and oral cavity samples. This clade contains the Firmicutes phylum, which are well-known members of the gut and oral microbiome [62].

Finally, the seventh selected Haar-like coordinate (59781) strongly localizes nostril samples. This clade consists entirely of the Corynebacterium genus, which is known to be a dominant bacteria in the nose [63].

**Fig 6:**
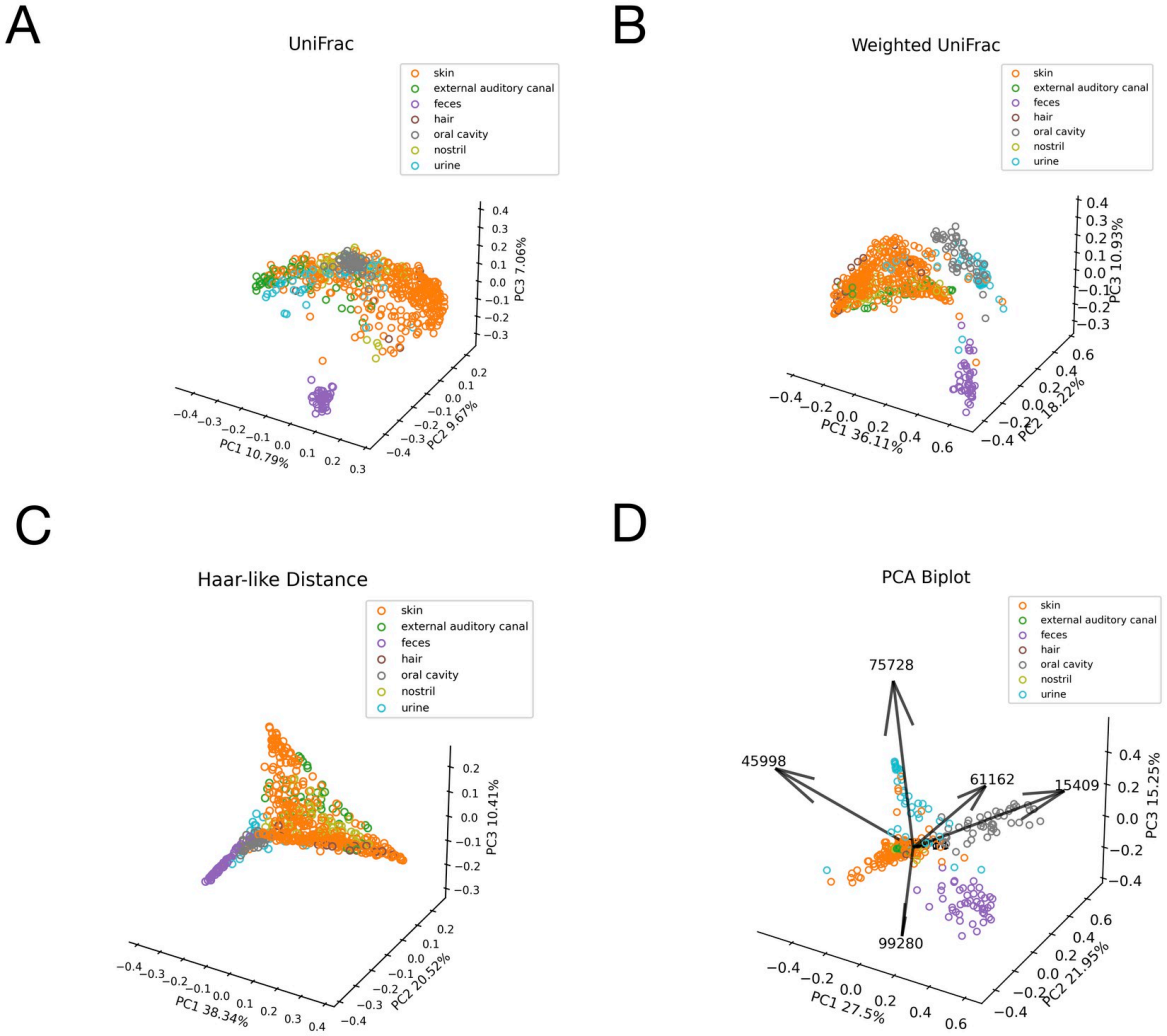
Comparison of the adaptive Haar-like embedding to various phylogenetic *β*-diversity metrics in the Body Sites dataset. Pseudo F-statistics are reported to quantify clustering. A: Unweighted UniFrac PCoA embedding (F = 23.51). B: Weighted UniFrac PCoA embedding (F = 88.24). C: Haar-like Distance PCoA embedding (F = 56.24). D: Adaptive Haar-like PCoA embedding using 7 Haar-like coordinates (F = 146.87).

**Fig 7:**
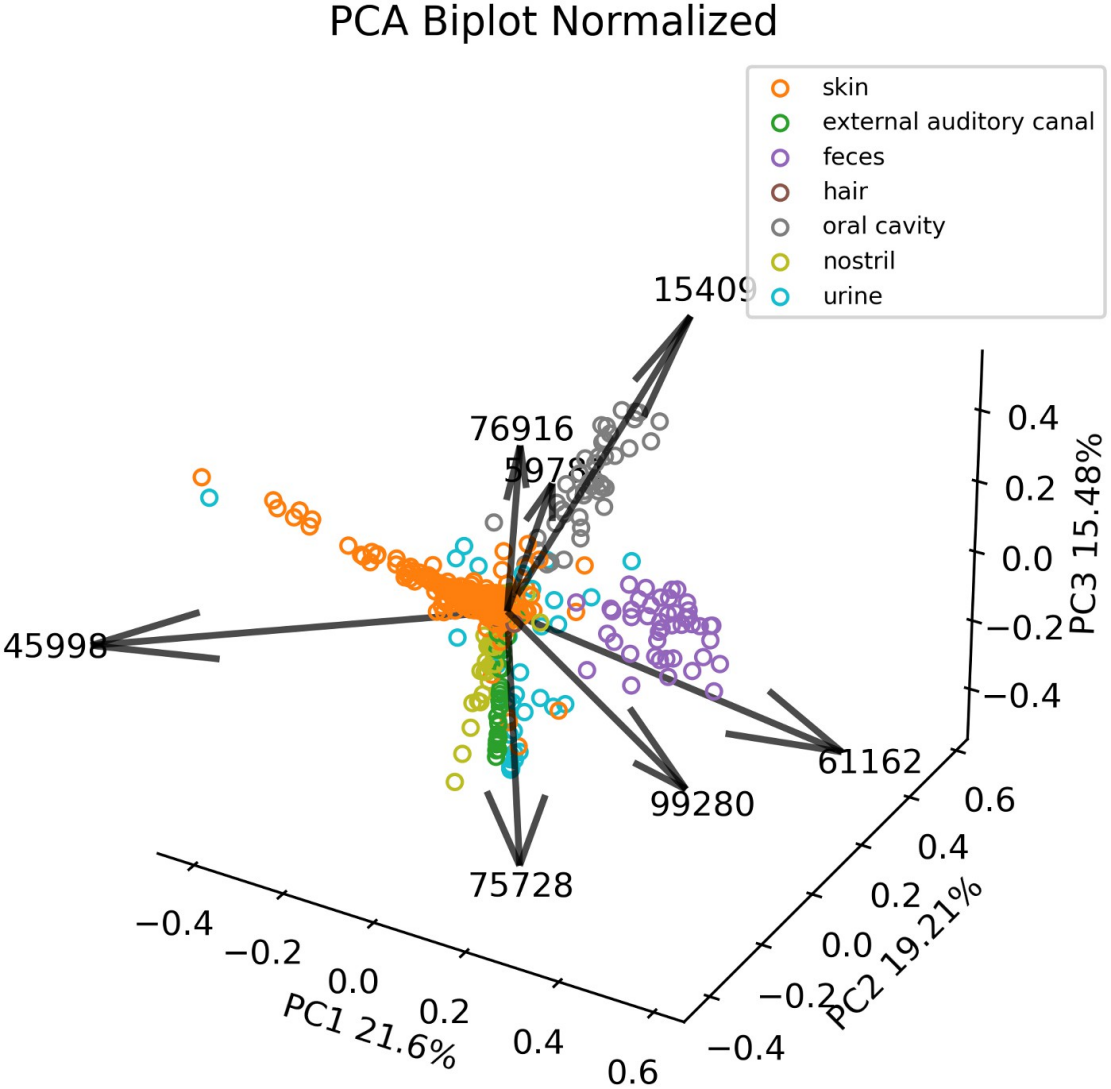
Normalized PCA of adaptive Haar-like distance using seven coordinates in the Body Sites dataset.

Based upon these top seven Haar-like coordinates, we then apply principal component analysis (PCA) to reduce to three dimensions for visualization. Comparing our PCoA embedding (Fig 6D) to the PCoA embeddings associated with unweighted UniFrac, weighted UniFrac, and the Haar-like distance (Fig 6A–6C) we see that our metric obtains better clustering by bodysite. Because this dataset has a large number of classes, it can be difficult to see all of the class separations in the biplot. For this reason, we also display the **normalized** PCoA embedding, resulting from a rescaling of the Haar-like coordinates, in Fig 7. In the normalized biplot, we can see all seven loadings, and it is clear that our metric is recovering Haar-like coordinates that align well (either in the positive or negative direction) with different classes in the embedding. For example, coordinate 15409, which was linked to Supragingival plaque, points exactly in the direction of significant variation for the oral cavity.

We can quantify how well each of the four metrics cluster the body habitats by the PERMANOVA pseudo-F test statistic [64] applied to the corresponding distance matrices. This score is a measure of clustering strength estimated by comparing within-group variability to between-group variability; a higher value indicates stronger clustering. As seen in Fig 6, the embedding associated with the adaptive Haar-like distance has the highest score and, by this measure, recovers the best clustering of body habitats among the various phylogenetic *β*-diversity metrics considered.

Altogether, only seven adaptive Haar-like coordinates are enough to cluster all body habitats except for the hair samples, which, as we have mentioned, represent a too small fraction of the dataset for localization with only seven Haar-like coordinates. Though separating body habitats may be a relatively trivial classification task, our method recovers coordinates that align almost perfectly with the various body habitats and outperforms existing metrics using just a few coordinates.

Next, we show that our algorithm maintains strong performance even in classification tasks deemed far more challenging by current metagenomic analyses.

### Dataset 2 classification: Autism

We turn our attention to the 16S dataset from “Altered gut microbial profile is associated with abnormal metabolism activity of Autism Spectrum Disorder” [46]. This study compared fecal samples from 143 individuals diagnosed with autism spectrum disorder (ASD) against 143 control subjects, matched for age and gender.

**Fig 8:**
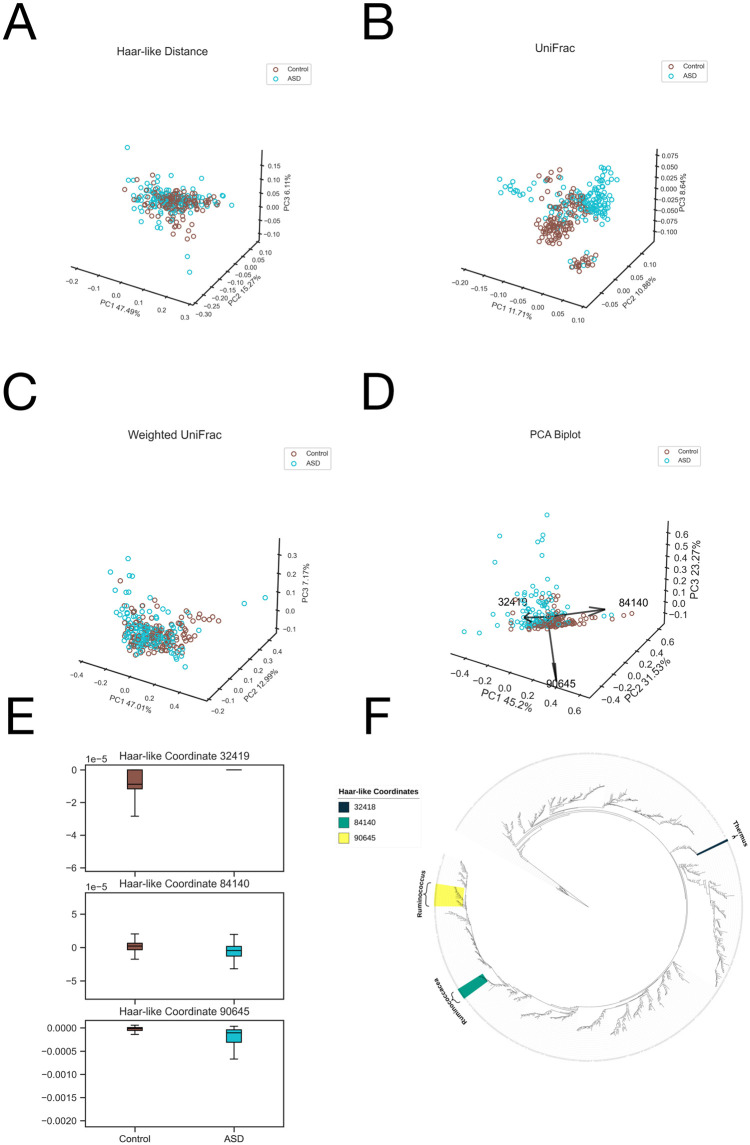
Comparison of the adaptive Haar-like embedding to various phylogenetic *β*-diversity metrics in the Autism dataset. A: Haar-like Distance PCoA embedding (F = 6.66). B: Unweighted UniFrac PCoA embedding (F = 18.44). C: Weighted UniFrac PCoA embedding (F = 7.80). D: Adaptive Haar-like PCoA embedding using 2 Haar-like coordinates (F = 34.96). E: Box plots of the top two Haar-like coordinates across the various diet types. F: The three most important Haar-like coordinates of the Autism dataset visualized on Greengenes 97%.

As seen in Fig 8E, the dominant Haar-like coordinate (32419) strongly distinguishes the ASD patients. This clade consists entirely of the genus Thermus, which has been observed to differ significantly in ASD patients compared to controls [65]. For the next prominent coordinate (84140), the associated clade is made up of an unclassified genus within the Ruminococcaceae family. Finally, for the third coordinate (90645), every member of the corresponding clade is classified as Ruminococcus, a genus that has been associated with ASD in the original study of this dataset [46], as well as in other studies [66–68].

In the phylogenetic spectrogram (Fig 8F), we note that the second and third coordinates (84140 and 90645) are more closely related (both belonging to the order Clostridiales) than the dominant coordinate (32419), which descends from Deinococci, a class of extremophiles. The role of these extremophiles, such as Thermus, in Autism spectrum disorder is not well understood, yet our methodology highlights their potential significance in this context.

For Dataset 2, we only compute the embedding associated with the three most dominant Haar-like coordinates. In the biplot (Fig 8D), we see that coordinates 90645 and 32419 are nearly orthogonal: 90645 captures the variation in ASD patients, while 32148 corresponds to control patients. However, we do not achieve the same quality of class separation as the previous dataset. This should be expected because the Body Sites data (Dataset 1) compared samples from distinct body habitats, which are known to harbor different microbial communities [45], while this dataset contains samples from the same habitat (feces). Consequently, it may be harder to find features that strongly distinguish the two groups. Regardless, the adaptive metric still achieves the best clustering among the tested metrics as indicated by the PERMANOVA statistics (Fig 8A–8D). This improvement in clustering compared to the (non-adaptive) Haar-like distance serves as compelling evidence for the role of weight optimization over the Haar-like coordinates to capture relevant differences in microbial composition.

Next, we apply our method on a WGS dataset where existing phylogenetic *β*-diversity metrics are unable to discern any clustering.

### Dataset 3 classification: Animal diet type

In this section, we analyze WGS data obtained from “Large scale metagenome assembly reveals novel animal-associated microbial diversity” [47]. This study compares 628 gut microbiomes from wild and captive animals “spanning 5 classes: Mammalia, Aves, Reptilia, Amphibia, and Actinopterygii.” We consider the diet types of these animals: carnivore, insectivore, omnivore, or herbivore.

**Fig 9:**
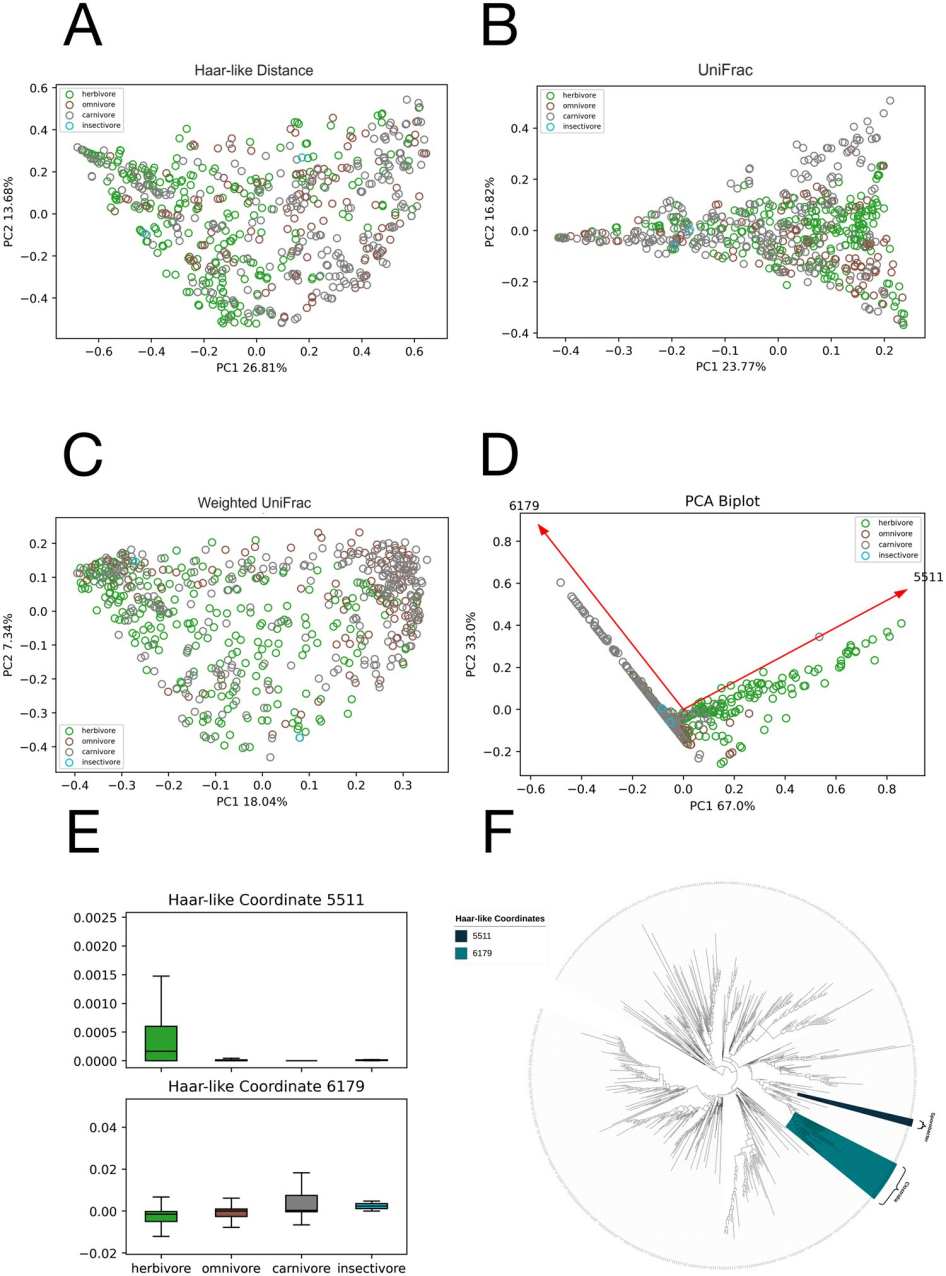
Comparison of the adaptive Haar-like embedding to various phylogenetic *β*-diversity metrics in the Animal Diet Type dataset. A: Haar-like Distance PCoA embedding (F = 9.35). B: Unweighted UniFrac PCoA embedding (F = 8.65). C: Weighted UniFrac PCoA embedding (F = 10.64). D: Adaptive Haar-like PCoA embedding using 2 Haar-like coordinates (F = 52.53). E: Box plots of the top two Haar-like coordinates across the various diet types. F: The two most important Haar-like coordinates of the Animal Diet dataset visualized on the WoL tree.

Training our model to recover just the two most important Haar-like coordinates, Fig 9E shows that the dominant coordinate (5511) strongly distinguishes herbivores from the other diet types. This coordinate consists of the genus sporobacter, which has been connected to various herbivores and ruminants [69–71]. The second coordinate (6179) increases in value moving from herbivore to omnivore to carnivore, with insectivore having similar values to carnivore. This coordinate contains members of the class Clostridia and, in particular, its left descendants (whose abundances contribute to a positive coordinate value) contain the order Clostridiales, which has been linked to some carnivorous species [72, 73]. As seen in the phylogenetic spectrogram (Fig 9F), these two Haar-like coordinates are closely evolutionarily related, both belonging to the Bacillota phylum.

Constructing the various *β*-diversity metric embeddings (Fig 9A–9D), we find that none of the traditional metrics recover any strong clustering or separation of the diet types. In contrast, the adaptive Haar-like embedding, using only two Haar-like coordinates, displays excellent separation of carnivore and herbivores, with omnivores laying directly in between the two.

We also note that our embedding allows for immediate visual identification of outliers. For example, there is one carnivore sample that is clustered closer to the herbivore samples. This sample corresponded to the European Grass snake and further investigation is necessary to determine if this is a general trend among this species or if this specific sample was an outlier.

Next, we demonstrate our methodology in a regression setting.

### Dataset 4 regression: Crohn’s disease

The first regression dataset we examine consists of WGS data from “Evaluating Metagenomic Prediction of the Metaproteome in a 4.5-Year Study of a Patient with Crohn’s Disease” [48]. A total of 8 fecal samples were collected from the patient over 5 years and processed in technical triplicate, resulting in a total of 24 samples. Additionally, various blood markers associated with inflammatory bowel disease were collected alongside these samples. Of these, “calprotectin was found to have the strongest association with the microbial dysbiosis index,” so we decided to train our model using the calprotectin value labels.

**Fig 10:**
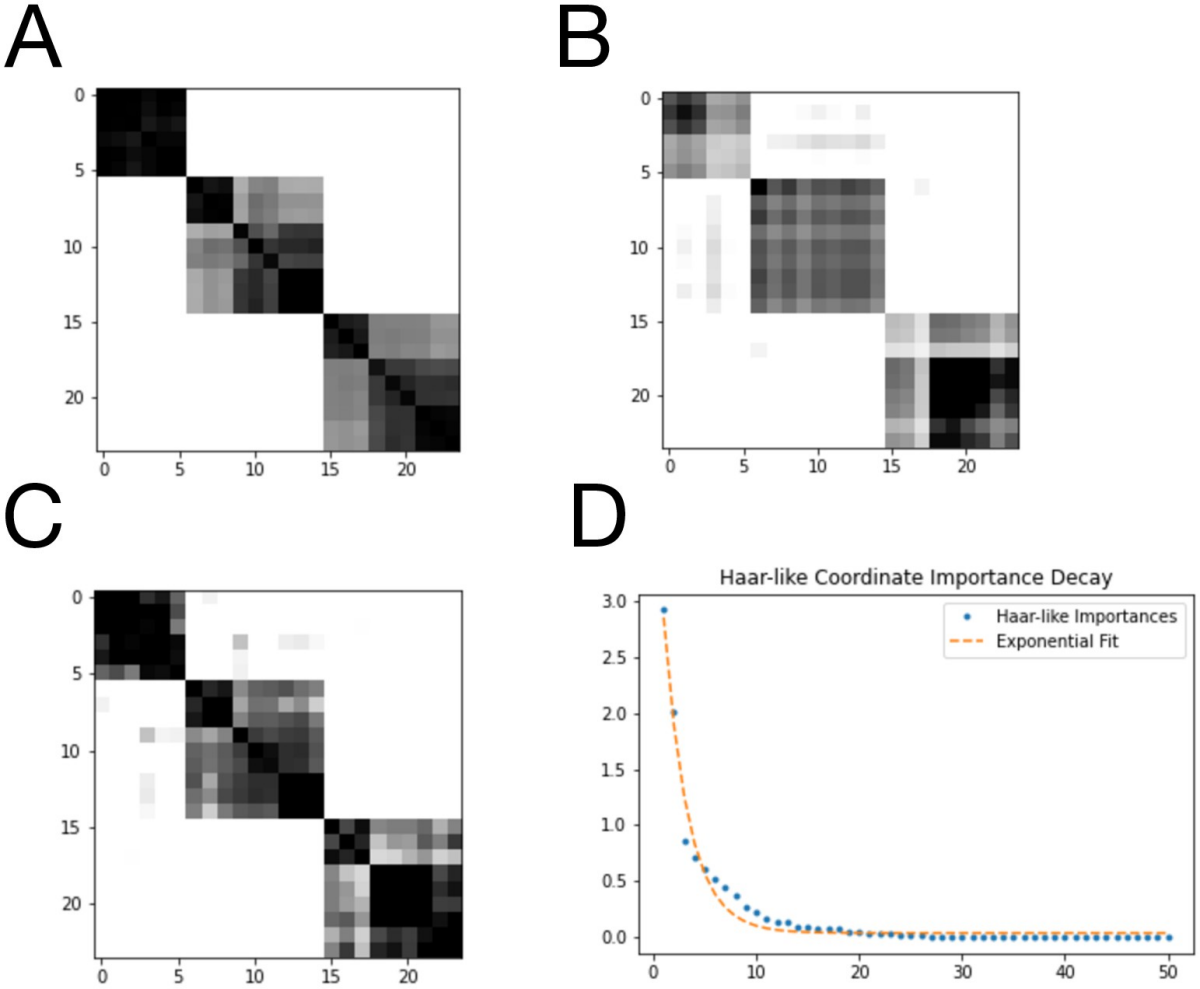
Sparse approximation of the RF Gram matrix from the Crohn’s dataset. A: RF Gram matrix. B: Sparse approximation using 2 Haar-like coordinates. C: Sparse approximation using 50 Haar-like coordinates. D: Haar-like coordinate importance as learned by Algorithm 1. The fit is *y* = 4.28*e*^−0.42*x*^ + 0.04.

Fig 10A displays the random forest Gram matrix. In the regression setting, we are ideally looking for a diagonal band in the matrix, indicating that samples with similar label values are highly similar. Here, we notice three main clusters corresponding to low, medium, and high calprotectin values, and an inner diagonal band of higher similarity. As seen in Fig 10B, just 2 Haar-like coordinates are sufficient to recover a similar clustering pattern to the original RF.

**Fig 11:**
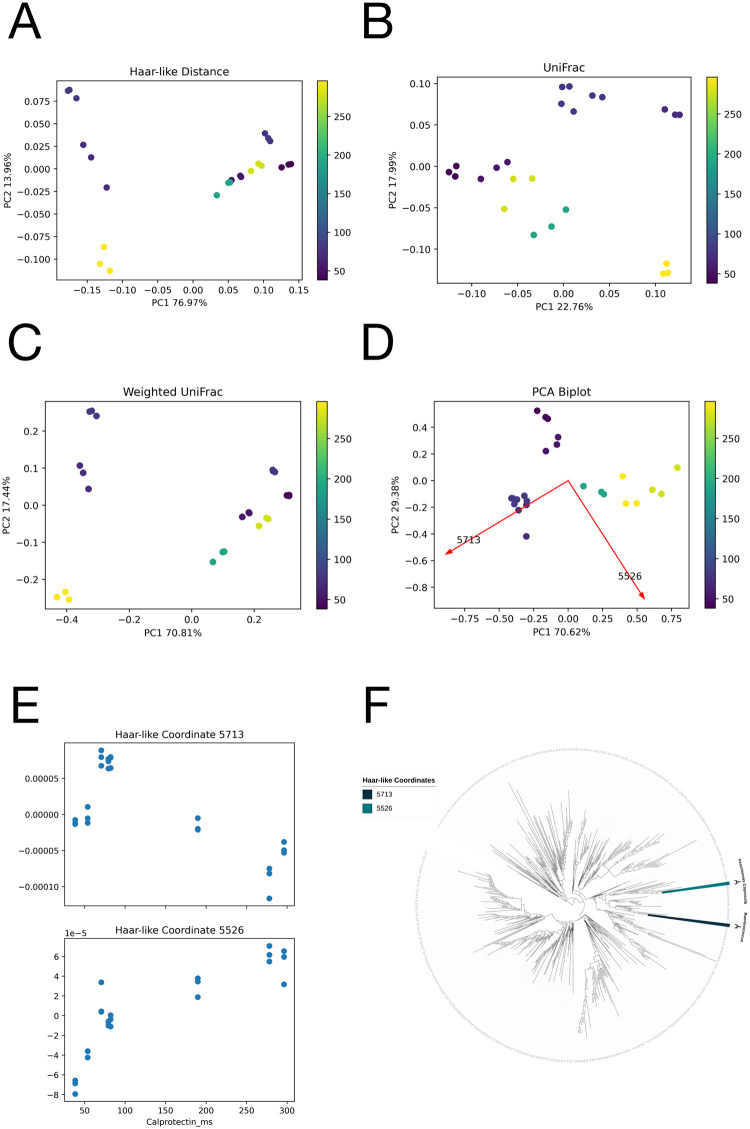
Comparison of the adaptive Haar-like embedding to various phylogenetic *β*-diversity metrics in the Crohn’s dataset. Distance correlations are reported to quantify gradients. A: Haar-like Distance PCoA embedding (Distance Correlation=.45). B: Unweighted UniFrac PCoA embedding (Distance Correlation=.67). C: Weighted UniFrac PCoA embedding (Distance Correlation=.45). D: Adaptive Haar-like PCoA embedding using 2 Haar-like coordinates (Distance Correlation=.91). E: Plots of the top two Haar-like coordinates across the samples. F: The two most important Haar-like coordinates of the Crohn’s dataset visualized on the WoL tree.

As seen in Fig 11E, the dominant Haar-like coordinate (5713) correlates negatively with Calprotectin levels. The corresponding clade consists of the genera Ruminococcus, Ruminococcus C, and Ruminococcus F. Multiple studies have associated these genera with Crohn’s disease and other gastrointestinal disorders [74–76]. Instead, the second Haar-like coordinate is positively correlated with Calprotectin levels and corresponds to the species Vescimonas coprocola. This species has been isolated from human feces [77] but is relatively unstudied, and the correlation observed here may warrant further scientific investigation into its relation to Chron’s disease.

To quantify the strength of the gradients in the regression setting, we use the notion of distance correlation [78]. As opposed to the traditional notion of correlation, distance correlation captures nonlinear associations, and a distance correlation of zero is equivalent to probabilistic independence. Among the traditional metrics, Unweighted UniFrac is the only one that displays any clear gradient with respect to calprotectin levels. Nevertheless, the adaptive Haar-like distance has the best gradient as quantified by distance correlation (see Fig 11A–11D).

Next, we demonstrate our metric on ocean sediment samples taken near an oil spill.

### Dataset 5 regression: Deepwater horizon oil spill

The final dataset we consider is a 16S dataset that “investigated the impact of oil deposition on microbial communities in surface sediments collected at 64 sites” affected by “the Deepwater Horizon oil spill in the spring of 2010” [49]. For our analysis, we consider each sample’s distance from the wellhead.

**Fig 12:**
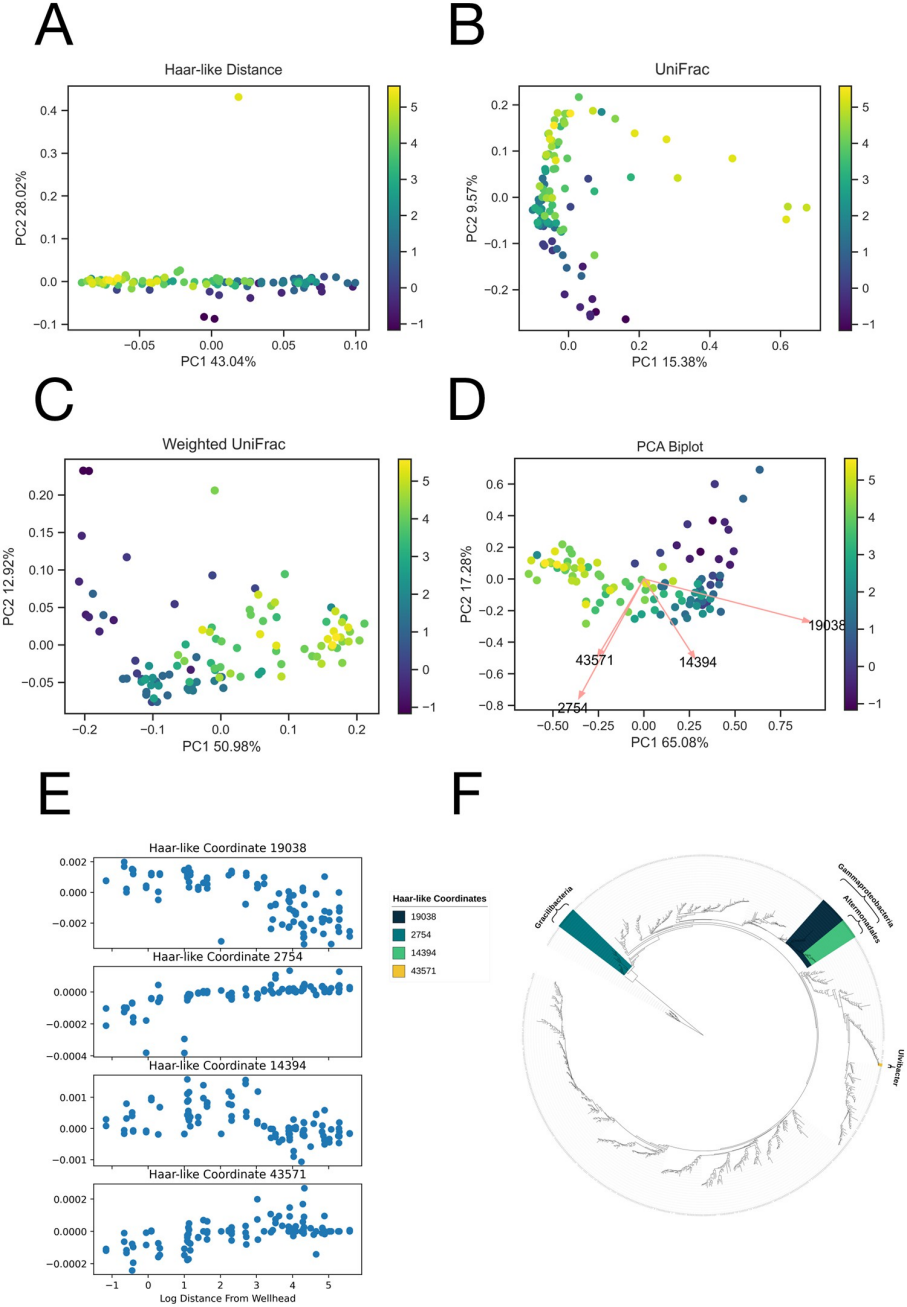
Comparison of the adaptive Haar-like embedding to various phylogenetic *β*-diversity metrics in the Deepwater Horizon dataset. A: Haar-like Distance PCoA embedding (Distance Correlation=.70). The outlier is associated with an OTU singleton. B: Unweighted UniFrac PCoA embedding (Distance Correlation=.60). C: Weighted UniFrac PCoA embedding (Distance Correlation=.70). D: Adaptive Haar-like PCoA embedding using 4 Haar-like coordinates (Distance Correlation=.72). E: Plots of the top four Haar-like coordinates across samples in the Deepwater Horizon oil spill dataset. F: The four most important Haar-like Coordinates visualized on Greengenes 97%.

In this dataset, the dominant Haar coordinate (19038) consists entirely of the class Gammaproteobacteria. As seen in Fig 12E, this Haar-like coordinate decreases with distance from the spill site, and as seen in Fig 12D, the corresponding loading aligns well with the distance gradient. This is consistent with the observation in the original publication [49], which noted that an uncultured Gammaproteobacterium OTU and a Colwellia taxon had high relative abundances in highly contaminated samples but low relative abundances elsewhere. The second Haar-like coordinate (2754) consists entirely of the phylum Gracilibacteria, which has been identified and examined in the context of oil spills previously [79]. The third Haar-like coordinate (14394) consists of the Alteromonadales order, which, as seen in Fig 12F, descends from the clade corresponding to the dominant coordinate (19038). Altermonadales abundance has also been identified and examined in oil-contaminated samples in [80]. Finally, the fourth Haar-like coordinate (43571) consists entirely of the genus Ulvibacter, which is known to be a hydrocarbon-degrading bacteria [81].

These learned Haar-like coordinates all correspond to clades that play a role in oil degradation, and as we show next, together, they are sufficient to cluster samples based on their distance from the wellhead accurately.

Constructing the adaptive Haar-like embedding using the top four Haar-like coordinates, we see a very clear gradient with respect to distance. For both the biplot (Fig 12D) and the plots of the individual Haar-like coordinates (Fig 12E), we take the logarithm of the sample distances in order to approximately linearize the distances (see Fig D in S1 Text). This has no effect on the resulting analysis and serves only for gradient visualization with respect to sample distance (to the wellhead) on a linear scale.

Finally, when comparing the distance correlation between the true wellhead distances and the various phylogenetic *β*-diversity metrics, we find that the adaptive Haar-like distance has the highest distance correlation among the four *β*-diversity phylogenetic metrics.

### Model validation

The preceding section highlighted how the adaptive Haar-like distance can generate insightful embeddings across diverse datasets. However, ensuring that the weights and coordinates derived from our metric closely match the RF estimates is imperative for the precise categorization of environmental attributes.

Here, we test the adaptive Haar-like kernel obtained from the microbiome learning repository (ML Repo) [50]. This repository consists of 28 binary classification problems and 5 regression tasks across various studies involving human microbial samples. We emphasize that in the framework of our model, classification is just another form of regression; hence, for simplicity, we chose to analyze only the binary classification problems.

Many of these datasets included only RefSeq OTU counts [82] and lacked Greengenes OTU counts. Due to the absence of an associated phylogenetic tree with RefSeq, these datasets were not suitable for our method. We also excluded datasets with sample size *n* ≤ 10 as well as datasets with extreme class imbalance (i.e., 25/75 split or worse). The remaining 16 datasets that we included in our analysis are detailed in Table 2.

**Table 2:** Datasets from ML Repo used for model comparisons. Acronyms “cd” and “uc” stand for Crohn’s disease and ulcerative colitis, respectively. Dataset names in the second column are as listed in [50].

Index	Dataset	Task	Sample Type	No. of Samples	Samples per Class
1	Montassier 2016	Bacteremia vs. no bacteremia	Human stool	28	11/17
2	David 2014	Animal vs. plant diet, last diet day	Human stool	18	9/9
3	Cho 2012	Chlortetracycline vs. control, cecal	Mouse cecal contents	17	7/10
4	Cho 2012	Chlortetracycline vs. control, fecal	Mouse pellets	18	8/10
5	Cho 2012	Penicillin vs. vancomycin, cecal	Mouse cecal contents	20	10/10
6	Cho 2012	Penicillin vs. vancomycin, fecal	Mouse pellets	19	9/10
7	Gevers 2014	Control vs. cd, ileum	Ileal biopsies	140	62/78
8	Gevers 2014	Control vs. cd, rectum	Rectal biopsies	160	92/68
9	Morgan 2012	Healthy vs. cd, stool	Human stool	81	19/62
10	Morgan 2012	Healthy vs. uc, stool	Human stool	66	19/47
11	HMP 2012	Male vs. female, stool	Human stool	180	98/82
12	HMP 2012	Stool vs. tongue	Human stool, oral	404	204/200
13	HMP 2012	Subgingival vs. supragingival plaque	Oral	408	203/205
14	Yatsunenko 2012	Malawi vs. Venezuela	Human stool	54	21/33
15	Yatsunenko 2012	Male vs. female	Human stool	129	37/92
16	Kostic 2012	Healthy vs. tumor biopsy, paired	Colon biopsies	172	86/86

To benchmark our method, we compare results to the original RFs and another state-of-the-art interpretable classifier, CoDaCoRe [44], that learns a sparse set of log-ratios to classify metagenomic data. We implemented a stratified 5-fold cross-validation (partitioning the data into 80% training and 20% testing) on each dataset, iterating this process with 5 different randomizations, resulting in 25 unique splits per dataset. In what follows, we outline the precise implementations of each model.

During the training of our adaptive Haar-like kernel, we employed a hyperparameter tuning stage to choose the optimal number of Haar-like coordinates for each dataset. For each randomization of the stratified 5-fold cross-validation, the 80% training data was further split into a training and hyperparameter selection set. The best-performing value of the parameter *s* was then chosen for the final model evaluation on the remaining 20% testing data.

Table 3 displays additional information about our model, namely the average and standard deviation of the number of coordinates selected, the top selected Haar-like coordinate in each dataset and a taxonomic classification that can be associated with that coordinate. The average *s* used for each dataset is reported in Table 3 as “Haar-like Sparsity.” We note that the optimal sparsity and its associated standard deviation depend strongly on the dataset. However, the overall low standard deviation observed across the datasets indicates that our model is relatively stable with respect to this parameter.

**Table 3:** Datasets from ML Repo used in our model comparisons. The “taxonomic classification” column lists the lowest taxonomic classification that encompasses all members of the clade corresponding to the given Haar-like coordinate. The phyla in dataset 12 include p_Actinobacteria, p_Firmicutes, and p_Tenericutes. Dataset names in the second column are as listed in [50].

Index	Dataset	CoDaCore sparsity	Haar-like sparsity	Top Haar-like coordinate	Taxonomic classification
1	Montassier 2016	n/a	7.12 ± 1.77	94036	o_Clostridiales
2	David 2014	n/a	6.56 ± 3.07	88556	o_Clostridiales
3	Cho 2012	n/a	2.16 ± 0.82	41082	f_S24–7
4	Cho 2012	n/a	1.00 ± 0.00	41082	f_S24–7
5	Cho 2012	n/a	2.28 ± 0.54	41082	f_S24–7
6	Cho 2012	n/a	6.56 ± 2.91	86819	o_Clostridiales
7	Gevers 2014	2.44	7.72 ± 1.87	86791	o_Clostridiales
8	Gevers 2014	2.40	5.88 ± 2.58	99273	f_Lachnospiraceae
9	Morgan 2012	1.76	3.16 ± 1.77	97006	f_Lachnospiraceae
10	Morgan 2012	1.36	4.48 ± 2.74	98982	f_Lachnospiraceae
11	HMP 2012	1.96	6.80 ± 2.13	38025	g_Bacteroides
12	HMP 2012	n/a	5.00 ± 0.00	99301	multiple phyla
13	HMP 2012	2.60	3.80 ± 1.22	79173	g_Parvimonas
14	Yatsunenko 2012	n/a	7.08 ± 1.72	84112	f_Ruminococcaceae
15	Yatsunenko 2012	1.88	6.84 ± 2.43	99190	f_Lachnospiraceae
16	Kostic 2012	1.32	7.88 ± 1.87	98472	g_Blautia

We observed better performance in our model by thresholding the Haar affinity matrix in (10). For this, we adopted the popular convention from K-Nearest Neighbors (KNN) classifiers and only kept the weights corresponding to the $\left\lfloor \sqrt{n}\right\rfloor$ closest neighbors. All other weights were set to zero.

For the RF classifier, we implemented the scikit-learn RF classifier [83] with the default parameter settings.

CoDaCoRe relies on a regularization parameter λ to control the trade-off between the sparsity and accuracy of the model. For a fair comparison with our model, we set λ = 0 to ensure the highest classification accuracy. The average model sparsity for each dataset is reported in Table 3 as “CoDaCoRe sparsity”. We note that for some datasets, especially those with a small number of samples, CoDaCoRe failed to find a fit due to perfect separation [84]. This occurs because of a logistic regression step in CoDaCoRe when an outcome variable entirely segregates a predictor variable, making it impossible to determine a regression coefficient. For these cases, the CoDaCoRe results were omitted (i.e., reported as n/a).

**Fig 13:**
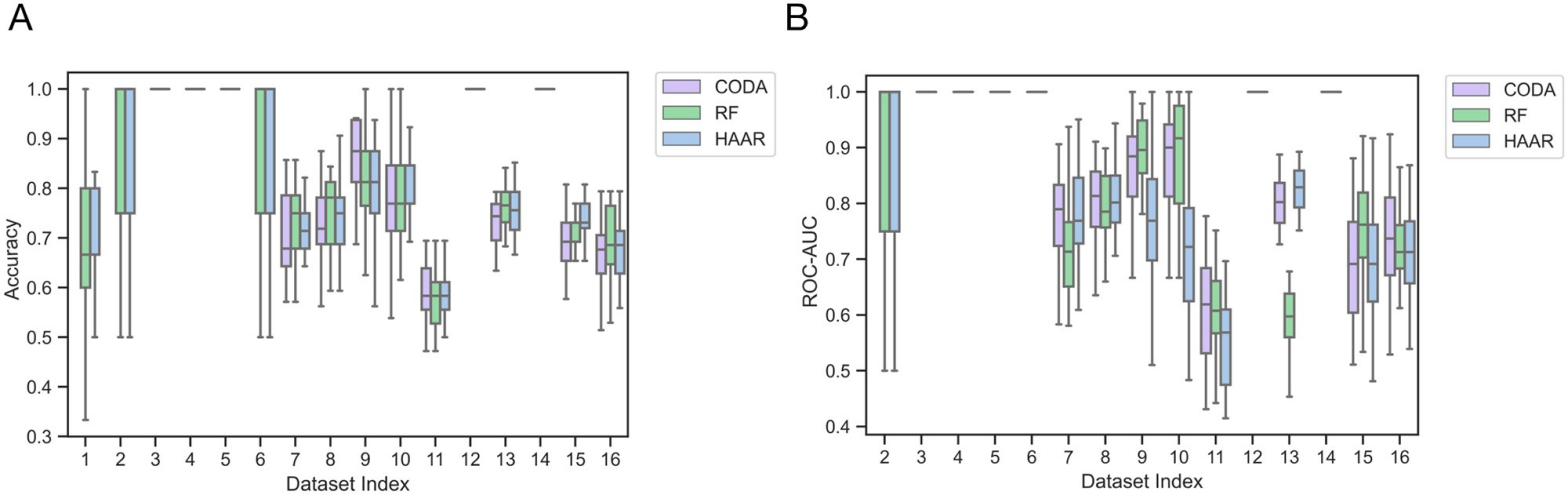
Model results across the 16 ML Repo datasets. A: Model accuracy. B: Model ROC-AUC scores.

Fig 13 displays boxplots of the accuracy and area under the receiver operating characteristic curve (ROC-AUC score) [85] for the adaptive Haar-like metric, CoDaCoRe, and RF across the sixteen datasets. Across all the tested datasets, we see that, on average, the three classification models are comparable in terms of accuracy. In terms of AUC, the adaptive Haar-like metric also has comparable performance except on the two datasets (Datasets 8–9 from Morgan 2012).

It is generally difficult to perform proper significance testing for model comparison via k-fold cross-validation because the assumption of independence is often violated, resulting in a high type-I error [86]. Regardless, in an attempt to quantify the significance of these results, we employed the heuristic corrected t-test described in [86]. With this corrected t-test and at a significance level of *α* =.05, we find that none of the differences in accuracy between RF and the adaptive Haar-like metric are statistically significant. Among the AUC values, only Dataset 9 from Morgan 2012 was found to have a significant difference where the RF outperformed our metric.

Initially, we expected RF to outperform the adaptive Haar-like distance on every dataset because RFs use non-stationary kernels that can fit higher-order interactions than adaptive Haar-like kernels (which are analogous to a Euclidean distance in a modified inner product space). However, because the adaptive Haar-like kernel can only learn linear relationships between microbial abundances, this may reduce over-fitting, allowing for comparable performance to RF in most datasets that we tested. On the other hand, while CoDaCoRe offers unrestricted OTU selection for crafting log-ratios, the adaptive Haar-like distance restricts OTU groupings based on phylogeny. Although this could enable CoDaCoRe to identify a more concise set of OTUs for model construction, we contend that restricting OTU grouping to the phylogeny enhances biological interpretability.

## Discussion

By learning a metric in a data-dependent manner, the adaptive Haar-like distance can produce accurate and insightful embeddings of metagenomic environments using only a limited number of Haar-like coordinates. The effectiveness of this approach hinges on the projection of compositional data into a wavelet basis that compares differences in abundance between the left- and right-clades that descend from each internal node in a reference phylogeny, which, up to a factor, is what each Haar-like coordinate represents. The sparsity induced by this choice of basis is precisely what allows our algorithm to learn a sparse set of weights that can approximate a far more intricate RF model. Analogous to wavelet denoising [87], selecting only the critical Haar-like coordinates in a dataset and discarding the rest helps build a robust representation of metagenomic environments, thus better differentiating genuine biological signals from noise. This is possible because of the one-to-one correspondence between the splits in the phylogeny and the wavelets.

As mentioned earlier, phylofactorization [32] uses a similar coordinate system to decompose microbial abundance in terms of internal nodes of a phylogeny. However, a pivotal distinction in our method lies in its **supervised** approach, where data labels are integrated to discern the most significant clades **within a specific setting**. In contrast, phylofactorization employs an **unsupervised** approach, reminiscent of PCA, to identify clades in the phylogeny that account for the most variance, **independently of data labels**.

We underscore that traditional statistical methods employed in Euclidean space do not apply to Haar-like coordinates. This distinguishes our approach from methods like phylofactorization or CoDaCoRe, which utilize isometric log-ratios and have established valid statistical tests [32]. Nonetheless, our method is the only one that exploits phylogenetic structure and takes a supervised learning approach. Further work is therefore necessary to derive statistical tests involving Haar-like coordinates.

Finally, it is worth noting that while we have introduced a data-driven approach for selecting the most significant Haar-like coordinates, our metric can also be applied to investigate user-specified Haar-like coordinate embeddings. Particularly, if specific clades hold particular scientific interest, our method can be used to generate biplots, thereby enabling the visualization of the Haar-like coordinates corresponding to particular clades.

## Conclusion

The adaptive Haar-like distance offers a versatile framework for comparing metagenomic samples from experiments encompassing various biological settings. By tailoring the underlying assumptions to each dataset, our metric learns weights on a reference phylogeny that best differentiate between environmental characteristics of interest. Compared to existing phylogenetic *β*-diversity metrics, the adaptive Haar-like distance can produce quantitatively better embeddings using only a handful of Haar-like coordinates. Our subsequent analysis of the Haar-like coordinates selected in each of the presented datasets confirmed that our metric learning algorithm recovers biologically meaningful splits in the phylogeny. This highlights using our metric as an exploratory tool for uncovering possible relationships between microbial clade abundances and environmental factors. Furthermore, by using the simple adaptive Haar-like kernel to approximate the patterns learned by a more complex but uninterpretable random forest, we offer an interpretable surrogate model with comparable performance.

## Supporting information

S1 Text The supplementary file contains the following figures and captions: **Fig A.** **Sparse approximation of the RF Gram matrix from the Autism dataset.** A: RF Gram matrix. B: Sparse approximation using 3 Haar-like coordinates. C: Sparse approximation using 50 Haar-like coordinates. D: Haar-like coordinate importance as learned by Algorithm 1. The fit is *y* = 5.73*e*^−0.13*x*^ + 1.21. **Fig B.** **Sparse approximation of the RF Gram matrix from the Animal Diet Type dataset.** A: RF Gram matrix. B: Sparse approximation using 2 Haar-like coordinates. C: Sparse approximation using 50 Haar-like coordinates. D: Haar-like coordinate importance as learned by Algorithm 1. The fit is *y* = 35.11*e*^−.55*x*^ + 2.11. **Fig C.** **Sparse approximation of the RF Gram matrix from the Deepwater Horizon oil spill dataset.** A: RF Gram matrix. B: Sparse approximation using 4 Haar-like coordinates. C: Sparse approximation using 50 Haar-like coordinates. D: Haar-like coordinate importance as learned by Algorithm 1. The fit is *y* = 16.00*e*^−0.61*x*^ + 0.64. **Fig D.** **Logarithm of sample distances from the wellhead in the Deepwater Horizon oil spill dataset.** **Fig E.** **Comparison of the KeRFE to a classifier constructed using its Euclidean approximation across the 16 datasets from the ML Repo datasets.** The comparison reveals no significant difference in accuracy between the two models.
(PDF)
