# Supplementary material for: Interpretable metric learning in comparative metagenomics: The adaptive Haar-like distance
Source: PLoS Comput Biol. 2024 May 20;20(5):e1011543. doi: 10.1371/journal.pcbi.1011543 (PMC11142682; doi:10.1371/journal.pcbi.1011543)
Supplement: S1 Text — The supplementary file contains the following figures and captions: Fig A. Sparse approximation of the RF Gram matrix from the Autism dataset. A: RF Gram matrix. B: Sparse approximation using 3 Haar-like coordinates. C: Sparse approximation using 50 Haar-like coordinates. D: Haar-like coordinate importance as learned by Algorithm 1. The fit is y = 5.73e−0.13x + 1.21. Fig B. Sparse approximation of the RF Gram matrix from the Animal Diet Type dataset. A: RF Gram matrix. B: Sparse approximation using 2 Haar-like coordinates. C: Sparse approximation using 50 Haar-like coordinates. D: Haar-like coordinate importance as learned by Algorithm 1. The fit is y = 35.11e−.55x + 2.11. Fig C. Sparse approximation of the RF Gram matrix from the Deepwater Horizon oil spill dataset. A: RF Gram matrix. B: Sparse approximation using 4 Haar-like coordinates. C: Sparse approximation using 50 Haar-like coordinates. D: Haar-like coordinate importance as learned by Algorithm 1. The fit is y = 16.00e−0.61x + 0.64. Fig D. Logarithm of sample distances from the wellhead in the Deepwater Horizon oil spill dataset. Fig E. Comparison of the KeRFE to a classifier constructed using its Euclidean approximation across the 16 datasets from the ML Repo datasets. The comparison reveals no significant difference in accuracy between the two models. (PDF) [file pcbi.1011543.s001.pdf]

Supporting Information

"Interpretable Metric Learning in Comparative Metagenomics: The Adaptive Haar-like Distance," by E. D. Gorman and M. E. Lladser

S1 Text

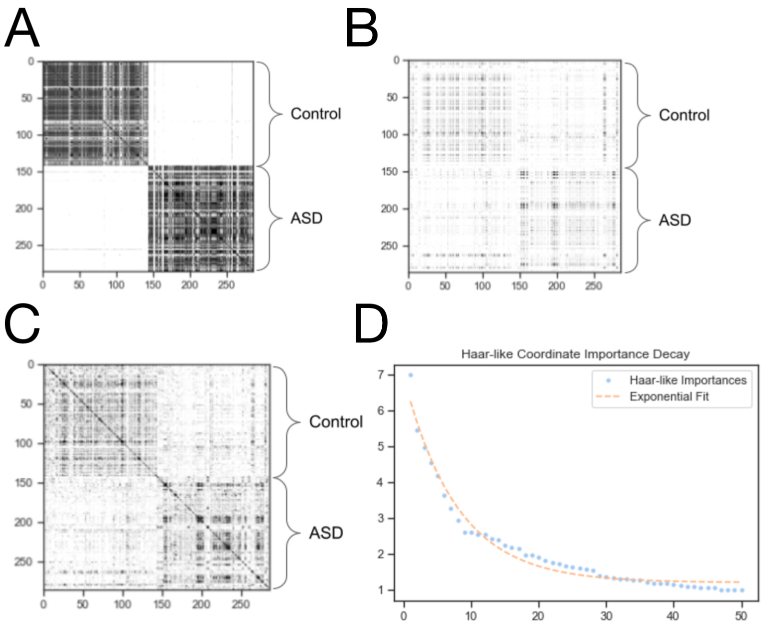

Fig A. Sparse approximation of the RF Gram matrix from the Autism dataset. A: RF Gram matrix. B: Sparse approximation using 3 Haar-like coordinates. C: Sparse approximation using 50 Haar-like coordinates. D: Haar-like coordinate importance as learned by Algorithm 1. The fit is $y = 5.73e^{-0.13x} + 1.21$.

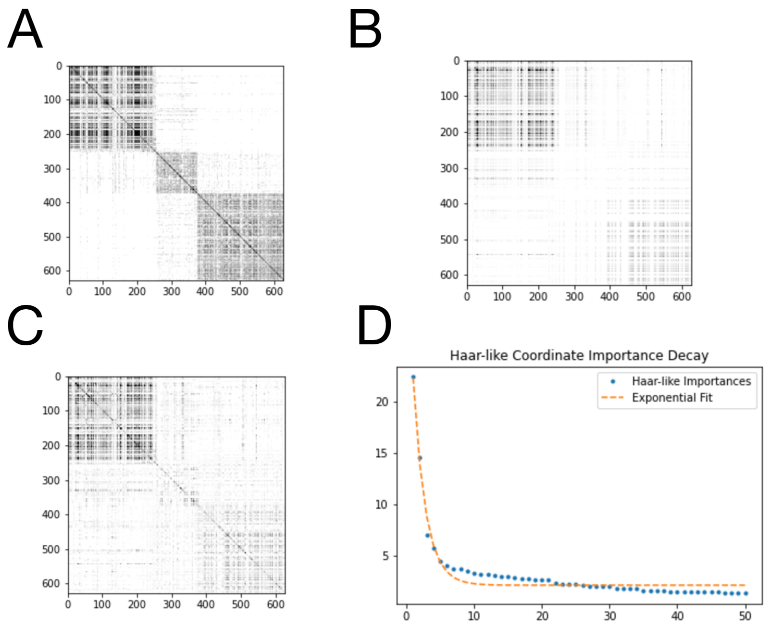

Fig B. Sparse approximation of the RF Gram matrix from the Animal Diet Type dataset. A: RF Gram matrix. B: Sparse approximation using 2 Haar-like coordinates. C: Sparse approximation using 50 Haar-like coordinates. D: Haar-like coordinate importance as learned by Algorithm 1. The fit is $y = 35.11e^{-.55x} + 2.11$.

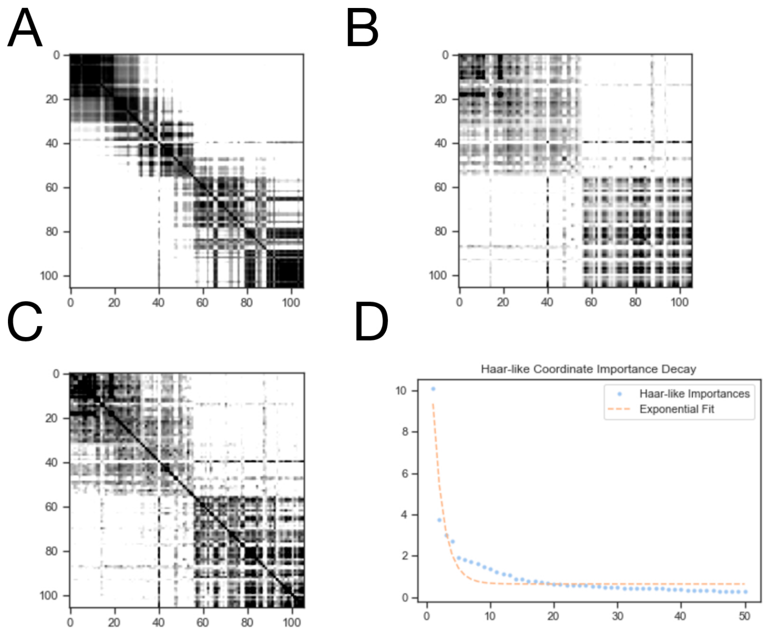

Fig C. Sparse approximation of the RF Gram matrix from the Deepwater Horizon oil spill dataset. A: RF Gram matrix. B: Sparse approximation using 4 Haar-like coordinates. C: Sparse approximation using 50 Haar-like coordinates. D: Haar-like coordinate importance as learned by Algorithm 1. The fit is $y = 16.00e^{-0.61x} + 0.64$.

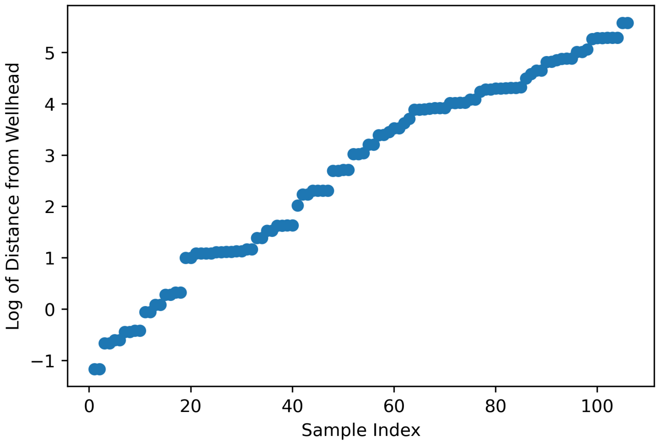

Fig D. Logarithm of sample distances from the wellhead in the Deepwater Horizon oil spill dataset.

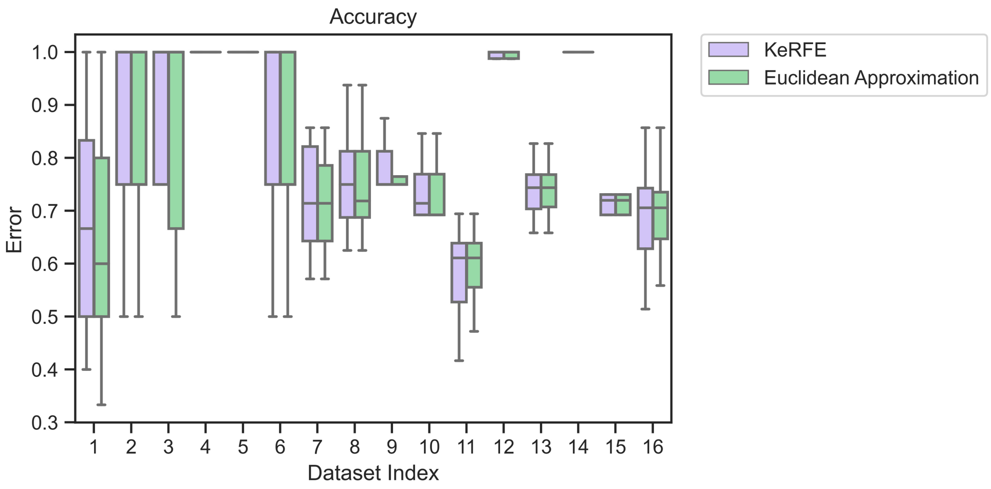

Fig E. Comparison of the KeRFE to a classifier constructed using its Euclidean approximation across the 16 datasets from the ML Repo datasets. The comparison reveals no significant difference in accuracy between the two models.
